# Recruited Cells Can Become Transformed and Overtake PDGF-Induced Murine Gliomas *In Vivo* during Tumor Progression

**DOI:** 10.1371/journal.pone.0020605

**Published:** 2011-07-06

**Authors:** Elena I. Fomchenko, Joseph D. Dougherty, Karim Y. Helmy, Amanda M. Katz, Alexander Pietras, Cameron Brennan, Jason T. Huse, Ana Milosevic, Eric C. Holland

**Affiliations:** 1 Department of Cancer Biology and Genetics, Memorial Sloan Kettering Cancer Center, New York, New York, United States of America; 2 Departments of Surgery (Neurosurgery) and Neurology, Memorial Sloan Kettering Cancer Center, New York, New York, United States of America; 3 Department of Molecular Biology, Rockefeller University, New York, New York, United States of America; 4 Departments of Human Oncology, Pathology and Pathogenesis, Memorial Sloan Kettering Cancer Center, New York, New York, United States of America; 5 Brain Tumor Center, Memorial Sloan Kettering Cancer Center, New York, New York, United States of America; The University of Chicago, United States of America

## Abstract

**Background:**

Gliomas are thought to form by clonal expansion from a single cell-of-origin, and progression-associated mutations to occur in its progeny cells. Glioma progression is associated with elevated growth factor signaling and loss of function of tumor suppressors *Ink4a*, *Arf* and *Pten*. Yet, gliomas are cellularly heterogeneous; they recruit and trap normal cells during infiltration.

**Methodology/Principal Findings:**

We performed lineage tracing in a retrovirally mediated, molecularly and histologically accurate mouse model of hPDGFb-driven gliomagenesis. We were able to distinguish cells in the tumor that were derived from the cell-of-origin from those that were not. Phenotypic, tumorigenic and expression analyses were performed on both populations of these cells. Here we show that during progression of hPDGFb-induced murine gliomas, tumor suppressor loss can expand the recruited cell population not derived from the cell-of-origin within glioma microenvironment to dominate regions of the tumor, with essentially no contribution from the progeny of glioma cell-of-origin. Moreover, the recruited cells can give rise to gliomas upon transplantation and passaging, acquire polysomal expression profiles and genetic aberrations typically present in glioma cells rather than normal progenitors, aid progeny cells in glioma initiation upon transplantation, and become independent of PDGFR signaling.

**Conclusions/Significance:**

These results indicate that non-cell-of-origin derived cells within glioma environment in the mouse can be corrupted to become *bona fide* tumor, and deviate from the generally established view of gliomagenesis.

## Introduction

Gliomas are the most common primary brain tumors, characterized by the infiltration of neighboring brain structures and robust expansion during progression to a *glioblastoma multiforme* (GBM) [1], [2]. Gliomas are frequently characterized by dysregulated signaling downstream of growth factor receptors such as EGFR, PDGFR, and IGFR, and elevated production of their corresponding ligands [3]–[6]. Nearly 30% of human gliomas show expression patterns that are correlated with PDGFR signaling [7], a pattern with prominent expression of *OLIG2* and other genes involved in CNS development referred to as “proneural” [8]. PDGF ligands (A–D) are upregulated in at least a third of surgical glioma samples and human glioma cell lines [9]–[13]. The importance of PDGF signaling is underscored in genetically engineered rodent gliomas, where overproduction of human PDGFb ligand is sufficient to induce gliomagenesis in a dose-dependent manner and allows to recapitulate the histologic, etiologic and pathobiologic character of the PDGF subset of human gliomas [14], [15]. Additionally, infusion of PDGF into the ventricles induces proliferation of the SVZ, resulting in lesions with some characteristics of gliomas [16]. Similar to human gliomas, mouse gliomas are cellularly and molecularly heterogeneous.

Glioma progression in humans is associated with deletion of the *INK4A*/*ARF* locus and loss of *PTEN* expression resulting in activation of Akt [3]–[6], [17]. The standard view of gliomagenesis is that sequential mutations occur and accumulate in cells derived from the glioma cell-of-origin. Indeed, many surgical GBM samples in patients appear clonal, with all tumor cells seemingly derived from the same cell; however, this may not necessarily mean they are derived from the cell-of-origin [18]–[21]. Cellular heterogeneity and reports of human gliomas comprised of several genetically unrelated clones suggest the possibility of oncogenic transformation in cells not derived from the glioma cell-of-origin [21]–[26]. The interconversion between human glioma subtypes upon recurrence and the existence of recurrent gliomas that lack mutations or deletions found in the original tumor further indicate the possibility for an expansion of an aggressive clone not arising from the cell-of-origin [8], [27]. In fact, PDGF-induced gliomas arising in both adult and neonatal rats have been shown to contain normal stem and progenitor cells “recruited” into glioma mass and induced to proliferate, indicating that proliferative stem-like portions of the tumor can arise from normal progenitors. However, the precise nature and specific functional characteristics of these “recruited” stem or progenitor cells have not been described.

Genetic analysis of surgical samples of human gliomas merely provides retrospective static information with regards to tumor evolution; lineage tracing from the cell-of-origin cannot be done in humans. Moreover, identifying and distinguishing GBM cells from the surrounding stroma is not a trivial task - glioma cells are often defined histologically, demonstrating high mitotic indices, expression of stem or progenitor cell markers, abnormal global gene expression patterns, presence of genetic alterations, and the ability to serially transplant the disease [3], [28], [29]. To investigate cellular contributions and structural/functional characteristics of “recruited” cells in murine gliomas during tumor progression, we used RCAS/tv-a and the *bacTRAP* systems [30]–[32]. Defining tumor cells by histologic criteria, genetic analysis, global gene expression profiling and transplantation studies, we studied the clonality of mouse gliomas with respect to the cell-of-origin. Here we show that in murine gliomas induced by human PDGFb (hPDGFb), glioma progression can occur by expansion of the recruited cells, and that these cells unrelated to glioma cell-of-origin can be corrupted to become *bona fide* tumor.

## Results

### Murine gliomas contain a recruited cell population

It has been recently shown that gliomas induced in adult or neonatal rats by hPDGFb-expressing retroviruses contain stem or progenitor-like cells expressing neural markers, that are contributing to glioma mass and are induced to proliferate by glioma environment [15], [33]. However, the nature and fate of these cells not derived from the glioma cell-of-origin has not been extensively studied. While these cells proliferate and express immature markers, questions as to whether they are functionally important in glioma progression, remain dependent from the glioma cell-of-origin, and whether they represent *bona fide* tumor cells, have not been addressed. In order to study this phenomenon of cellular contribution to glioma heterogeneity, we employed lineage tracing, molecular analysis and functional characterization of non-cell-of-origin derived cells using two different mouse models of hPDGFb-driven gliomas.

**Figure 1:**
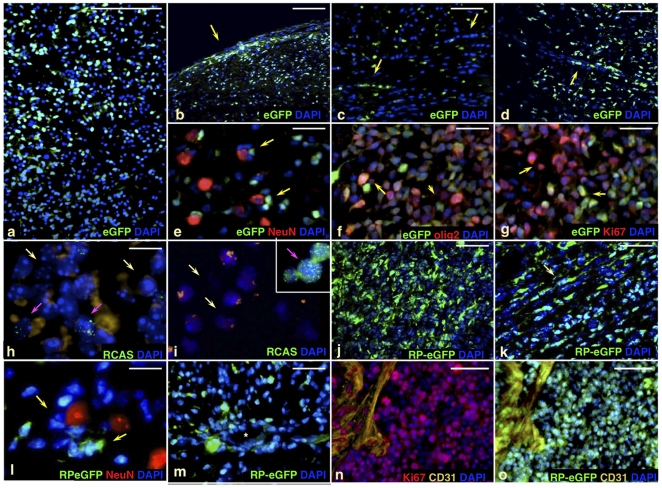
PDGF-driven murine gliomas contain recruited cells that are not derived from the cell-of-origin. (**a–g**) Virally encoded eGFP on DAPI-stained frozen sections of RCAS-PSG-induced wild-type murine *Ntv-a* gliomas. Yellow arrows in (**b–e**) show Scherer's secondary structures, including subpial accumulations of tumor cells (**b**), white matter tracking (**c**), perivascular accumulations of glioma cells (**d**), and perineuronal satelliting cells (**e**). Scherer structures are contributed to by both progeny and recruited cells. Sections in (**e**,**f,g**) were stained with NeuN, olig2, and Ki67, shown in red. Arrows in (**f,g**) indicate Ki-67 (**f**) or olig2 (**g**)-expressing progeny cells (yellow) and recruited cells (pink). (**h,i**), FISH analysis of murine glioma sections (**h**), FACS-sorted eGFP-negative recruited cells (**i**), and eGFP-positive sorted progeny cells (**i**, inset). Pink arrows in (**h**; **i** inset) indicate presence of multiple integrations in progeny cells (green signal); note the absence of integrations in recruited cells (white arrows; **h**,**i**). Note that the majority of cells in the tumor section appear recruited, and viral integration correlates with the eGFP expression by FACS. (**j–o**) Native RP-eGFP on DAPI-stained frozen sections of *bacTRAP* gliomas induced by orthotopic transplantation of non-fluorescent hPDGFb-driven primary mouse glioma cells into the *bacTRAP olig2 RP-eGFP* reporter mice. Yellow arrows in (**k,l**) indicate Scherer's secondary structures, containing RP-eGFP-positive recruited cells; asterisk in (**m**) shows the lumen of a large vessel. (**n,o**) Ki67 staining (red) and CD31 staining (yellow) indicate that most olig2 recruited cells expressing *RP-eGFP* in transplanted gliomas proliferate robustly.

We first infected *Nestin-tv-a* (*Ntv-a*) mice with an RCAS vector expressing hPDGFb and eGFP (RCAS-hPDGFb-HA-SV40-eGFP, “RCAS-PSG”) (**Figure 1a–i**) [14], [30], [34]. RCAS/tv-a is a retroviral system that allows for lineage tracing from the nestin-positive glioma cell-of-origin, and the use of this specific vector labels the cell-of-origin and its progeny with eGFP expression. Consistent with the previously published results from others and us, RCAS-PSG induced high- and low-grade gliomas with typical oligodendroglioma histology, containing proliferating cells expressing olig2 and other markers of neural cell lineages (**Figure 1f,g**; **Figure S1a–d; Figure S2c–f; Figure S3a**). Many but not all of these cells were derived from the cell-of-origin and expressed viral eGFP (“progeny cells”), while others appeared to not be infected with the RCAS-PSG virus (“recruited cells”), as they did not express eGFP or hPDGFb, with hPDGFb expression determined by immunostaining for hemagglutinin epitope tag included in RCAS-PSG vector (**Figure S2a–f**) [14]–[16], [33]. In comparison to the previously published RCAS-hPDGFb vectors, the levels of hPDGFb expression by RCAS-PSG were 3–4 fold lower (**data not shown**) [14]. We confirmed the correlation between eGFP expression and viral integration using RT-PCR (reverse transcription), real-time qPCR and FISH in murine glioma sections and eGFP-positive or eGFP-negative FACS-sorted glioma cell populations (**Figure 1h,i** **; Figure S1e,f**). In these tumors, histologically-defined pathognomic glioma structures (secondary structures of Scherer [35]) contained both progeny and recruited cells (**Figure 1b–e**; **Figure S1a–d**). We refer to these tumors as “virally-induced gliomas”.

Modeling systems employing intracranial transplantation of glioma cells are frequently used in glioma research. To rule out the possibility of spontaneous proviral excision or loss of eGFP expression in the originally infected cells in the virally induced gliomas, we performed transplantations of acutely isolated non-fluorescent hPDGFb-driven mouse glioma cells derived from Ntv-a gliomas induced by the non-fluorescent RCAS-hPDGFb vector into the *bacTRAP olig2 RP-eGFP* reporter mice, expressing a fusion between ribosomal protein L10a and eGFP (*RP-eGFP*) under the *Olig2* promoter (**Figure S3b–d**) [14], [31], [32]. In these “transplanted gliomas”, RP-eGFP-expressing host cells represent olig2-positive recruited cells, while transplanted glioma cells do not express eGFP. Similar to virally-induced gliomas, RP-eGFP-expressing host-derived recruited cells proliferated (stained for Ki-67), showed spindloid and/or typical oligodendroglial morphology, and contributed to the secondary structures of Scherer (**Figure 1j–o**). In virally-induced gliomas, olig2 progeny and olig2 recruited cells are strictly defined by the presence or absence of eGFP expression; in contrast, in transplanted gliomas, the injected non-fluorescent glioma cell population is a mixture of progeny and recruited cells. Thus, identification of recruited cells by RP-eGFP expression in this system likely underestimates the true contribution of recruited cells to the glioma mass. While virally-induced gliomas have been shown to approximate the human scenario for at least a subclass of human gliomas, the extent of similarity of transplanted gliomas to the patient tumors, as well as to other transplantation systems, has not been addressed [3]. An additional caveat of the *bacTRAP* system is that olig2 RP-eGFP expression in the adult murine brain labels not only progenitor cells, but also more differentiated oligodendrocytes [32].

### Tumor suppressor loss expands the recruited population

**Figure 2:**
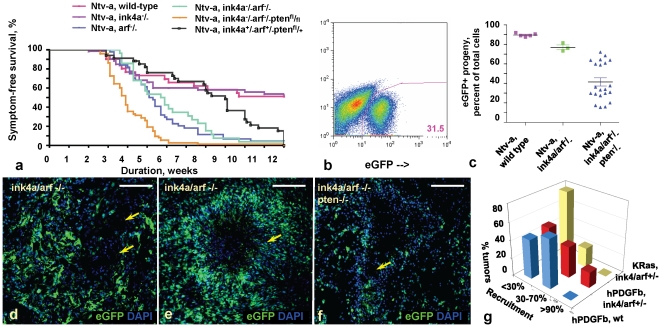
Homozygous tumor suppressor loss at glioma initiation enhances tumor formation and expands the recruited glioma cell fraction. (**a**) Kaplan-Meyer analysis for symptomatic gliomas in various *Ntv-a* mouse strains listed above the graph, injected with RCAS-PSG and RCAS-Cre, if applicable. Each curve represents survival analysis for n≥30 animals. (**b,c**) Quantification of percentages of progeny cells in *Ntv-a* gliomas, which are wild-type, deleted for *Ink4a/Arf*, or for *Ink4a/Arf* and *Pten*, as measured by FACS. Tumor suppressor loss expands the recruited component in mouse gliomas. (**d–f**) DAPI-stained images of frozen sections of murine gliomas, showing virally encoded eGFP in RCAS-PSG-induced Ntv-a GBMs deleted for *Ink4a/Arf* (d,e) or *Ink4a/Arf* and *Pten* (**f**). (**g**) Quantification of number of transplanted gliomas (z-axis) containing various percentages of recruited cells (x-axis), induced by transplantation of non-fluorescent hPDGFb-driven or Ras-driven murine glioma cells into the wild-type or *Ink4a/Arf^+/-^ bacTRAP olig2 RP-eGFP* hosts (y-axis). Note that significant recruitment occurs upon transplantation of either hPDGFb-driven and Ras-driven murine glioma cells into the *Ink4a/Arf^+/-^* hosts.

As human gliomas progress, loss of tumor suppressor function is seen in nearly all cases [6]. It is possible that loss of tumor suppressors in the progeny or the recruited populations during glioma progression could give a preferential growth advantage to either of these cell populations. Therefore, to investigate the relative effects of tumor suppressor loss on progeny and recruited cells, we induced gliomas in *Ntv-a* mice with germline deletions of *Ink4a*, *Arf* and/or *Pten*, the latter achieved by Cre-mediated recombination of the floxed *Pten* alleles by RCAS-Cre [36], [37]. As previously published, we found that loss of *Arf* and *Pten*, but not *Ink4a*, shortened tumor latency and increased incidence of murine GBMs, histologically defined by the presence of microvascular proliferation and pseudopalisading necrosis (**Figure 2a** **; Figure S4a,b**) [3], [36], [37]. Of note, the contribution of the recruited cells to high-grade glioma structures was most prominent in the *Ntv-a Ink4a/Arf* ^-/-^, *Pten* ^-/-^ tumors (**Figure 2b–f**).

We then determined whether such robust expansion of the recruited cell population upon tumor suppressor loss is limited to modeling systems characterized by paracrine growth factor signaling, or whether it could apply to murine gliomas with cell-autonomous activation of oncogenes. While mutations in *RAS* are not characteristic of a given human glioma subtype, we used overexpression of mutant *RAS* as a proxy to model human gliomas driven in a cell-autonomous manner [38]. For this purpose, we created RCAS-mRFP-SV40-Ras (RCAS-RSR) vector that expresses mutant *KRASG12D* and simultaneously marks the infected cells by expression of mRFP (**Figure S5a–e**). While this vector was not robust at glioma initiation (<10% incidence), injections of RCAS-RSR and RCAS-Cre into *Ntv-a Ink4a/Arf^-/-^Pten^fl/fl^* mice could give rise to high-grade gliomas that did not express PDGFRα and contained regions of proliferating cells that did not express mRFP (**Figure S5d,e,g**).

To further address the ability of the recruited cells characterized by tumor suppressor loss to contribute to high-grade glioma structures, we performed transplantation experiments using the *bacTRAP* system. Transplantation of non-fluorescent hPDGFb-driven glioma cells into *Ink4a/Arf^-/-^Pten^fl/fl^ bacTRAP olig2 RP-eGFP* reporter mice resulted in formation of high-grade glioma structures comprised of both progeny and recruited cells (**Figure S5h**). Transplanted gliomas induced by hPDGFb-driven glioma cells in hosts with altered tumor suppressor function showed higher percentages of the overall recruitment than in the wild-type hosts, indicating that complete or partial tumor suppressor loss may enhance the ability of the cells to be recruited (**Figure 2g**). Although gliomas induced by transplantation of non-fluorescent *Pten*-deleted Ras-driven murine glioma cells showed recruitment of proliferating host brain cells into the glioma mass (**Figure S5f,h**), the overall amount of recruited cells into Ras-driven tumors and the number of tumors that showed large regions of recruitment was significantly less than that seen with the hPDGFb-induced gliomas (**Figure 2g**).

### Recruited cells in hPDGF-induced can overtake glioma mass during progression

The above data indicates that murine glioma cells, especially those driven by PDGF signaling, recruit proliferating olig2-expressing progenitors, and their contribution to high-grade glioma structures is enhanced upon homozygous loss of *Ink4a/Arf* and *Pten* at glioma initiation. However, human gliomas undergo tumor suppressor loss stochastically as they progress. Stochastic tumor suppressor loss could in principle occur in any cell subjected to the pressures of glioma environment, regardless of its progeny or recruited status. To mimic this phenomenon, we used *Ntv-a Ink4a/Arf^+/-^Pten^+/fl^* mice infected with RCAS-PSG and RCAS-Cre. Given the constraints of our experimental system, formation of murine gliomas with high-grade architectural features would require loss of function of the remaining tumor suppressor allele, by either stochastic loss of heterozygosity (LOH) or epigenetic events. The rarity of such events would in turn result in the expansion of an altered cell population from a single cell, be it progeny or recruited. Although neither *Ntv-a Ink4a/Arf^+/-^Pten^+/fl^* nor *Ntv-a Ink4a/Arf^-/-^Pten^fl/fl^* mice generate spontaneous gliomas, it is likely that experimentally provided genetic hits may represent the rate limiting step for transformation and thus, transformation of recruited cells may be less common in human gliomas.

**Figure 3:**
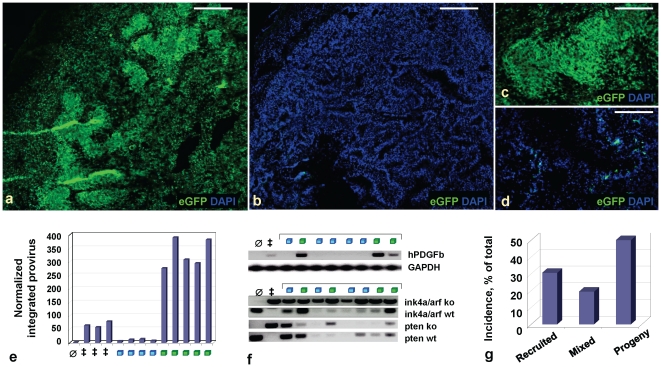
GBMs arising in mice targeted for heterozygous tumor suppressor loss show expansion of either recruited or progeny cell populations in pseudopalisade regions. DAPI-stained composite (**a,b**) and 20X (**c,d**) images of frozen sections showing virally encoded eGFP in RCAS-PSG-induced Ntv-a GBMs heterozygous for loss of *Ink4a*, *Arf* and *Pten* at tumor initiation, with stochastic tumor suppressor loss in regions of progeny (**a,c**) or recruited (**b,d**) cells during tumor progression. (**e**) real-time qPCR for viral hPDGFb-HA on tumor cell DNA extracted from mouse GBMs, indicating much lesser amounts of viral integrations in GBMs containing recruited pseudopalisades (blue squares), as compared to progeny pseudopalisades (green squares). Graph shows fold change over the background in the uninjected adult mouse brain (∅); all analyses are normalized to GAPDH as a control. ‡, positive control, RCAS-PSG Ntv-a GBMs deleted for *Ink4a/Arf* and *Pten*. (**f**) PCR for viral hPDGFb-HA, wild-type or knockout *Ink4a/Arf* loci and *Pten* alleles on tumor cell DNA extracted from mouse glioma sections. hPDGFb-HA PCR: ∅, negative control, uninjected adult mouse brain; ‡, positive control, Ntv-a GBM homozygous deleted for *Ink4a*, *Arf* and *Pten*. GAPDH PCR was done in the same tube. *Ink4a/Arf*, *Pten* PCR: ∅ and ‡ indicate controls for wild-type or knockout alleles and loci, respectively (presence of band in KO lane indicates deleted allele). Gliomas that lost *Ink4a/Arf* during tumor progression were also likely to lose wild-type *Pten* allele. (**g**) Incidence of GBMs containing regions of recruited cells, progeny cells or a mixture of these cells in pseudopalisade regions, in Ntv-a mice heterozygous targeted for loss of *Ink4a*, *Arf* and *Pten*. While most GBMs arising in mice heterozygous deleted for *Ink4a/Arf* and *Pten* at glioma initiation contain large regions of progeny, about ∼30% show large areas of recruitment (total number of tumors analyzed, n = 36).

While the latency of gliomas induced in *Ntv-a Ink4a/Arf* ^+/-^ *Pten* ^+/fl^ mice by RCAS-PSG and RCAS-Cre was intermediate between the wild type and *Ink4a/Arf* ^-/-^ *Pten* ^fl/fl^ mice, the distribution of glioma grades at the point when the tumors caused mice to become moribund was similar to that of gliomas characterized by homozygous tumor suppressor loss at initiation (**Figure 2a**; **Figure S4b**). However, unlike the mixed progeny-recruited cell composition seen in gliomas null for *Ink4a*, *Arf* and *Pten* at tumor initiation, gliomas arising in mice initially heterozygous for these tumor suppressors frequently showed regional domination by either progeny or recruited cells (**Figure 3a–d**). In the most extreme cases, such glioma regions were almost fully comprised of eGFP-negative recruited cells, as verified by FACS and qPCR (**Figure 3a,b,e** **; Figure S5j,k**). Of note, these regions dominated by recruited cells showed loss of function of the remaining wild type tumor suppressor alleles by LOH (in the case of *Ink4a/Arf*), or loss of expression at the protein level with or without LOH (in the case of *Pten*), as in human gliomas (**Figure 3f** **; Figure S4c–e**). While *Ink4a/Arf* loci were most frequently lost at the genetic level as determined by PCR, of 40 hPDGFb-driven gliomas arising in *Ntv-a Ink4a/Arf^-/-^Pten^fl/fl^* or *Ntv-a Ink4a/Arf^+/-^Pten^+/fl^* injected with RCAS-Cre, 28 did not express Pten; of these, 21 retained *Pten* allele by real-time PCR, while 7 lost it (**Figure S4c**). Thus, while loss of Pten expression occurred in ∼70% of all cases, loss of *Pten* at the DNA level occurred in 17.5% cases. There was a small but statistically significant difference with respect to latency medians depending on whether a given glioma was predominantly derived from progeny or recruited cells (two-tailed unpaired Student's t-test, p<0.006) (**Figure S5i**). However, their variances largely overlapped (F test), and GBMs containing large areas with pseudopalisades predominantly comprised of recruited cells represented ∼30% of all mouse GBM cases (**Figure 3g**).

### Recruited regions of hPDGF-induced gliomas can become independent of PDGF signaling

**Figure 4:**
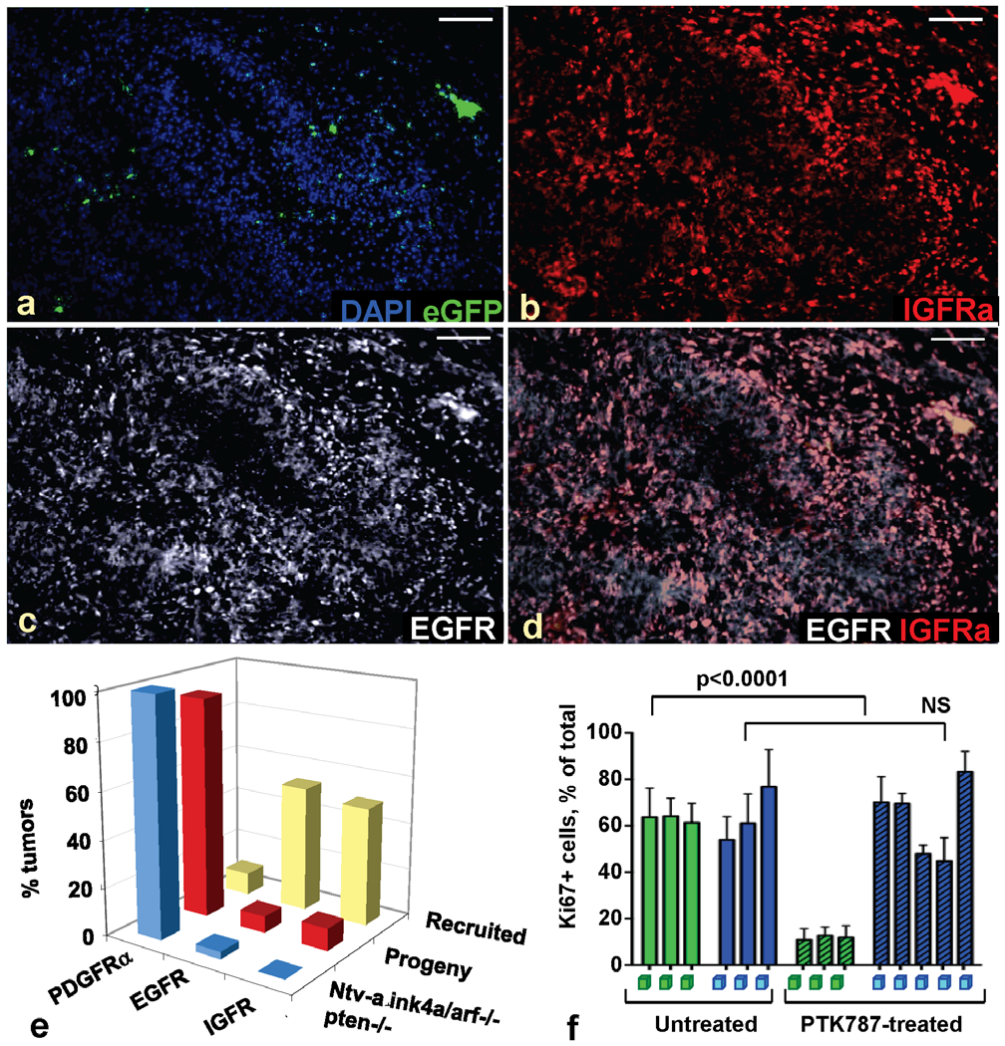
Glioma regions dominated by recruited cells can become independent of PDGFR signaling. (**a–d**) DAPI-stained frozen section of an *Ntv-a* RCAS-PSG-induced GBM heterozygous deleted for *Ink4a/Arf* and *Pten* at tumor initiation, containing large areas of recruitment expressing IGFR (**b**, red) and EGFR (**c**, white); (**d**) shows composite. (**e**) Quantification of growth factor receptor expression in *Ntv-a* GBMs in homozygous (n = 22) or heterozygous deleted backgrounds, containing large regions of progeny cells (“progeny”, n = 43) or recruited cells (“recruited” n = 32), by immunostaining. (**f**) Percent of Ki67-positive cells in untreated or PTK787-treated *Ntv-a* GBMs heterozygous deleted at glioma initiation and containing large regions of progeny (green squares) or recruited (blue squares) cells. Recruited cells in RCAS-PSG-induced *Ntv-a* GBMs heterozygous deleted for *Ink4a*, *Arf*, *Pten* at tumor initiation and containing large recruited areas do not reduce their proliferation rates in response to treatment with PDGFR/VEGFR inhibitor PTK787, suggesting that they become independent of PDGFR signaling.

We then characterized tumors with regions dominated by recruited cells for expression of growth factor receptors commonly activated in human gliomas, including PDGFR, EGFR and IGFR [3]–[5]. In gliomas where both alleles of *Ink4a, Arf, Pten* were deleted at tumor initiation, and in regions of gliomas dominated by progeny in tumors with initially heterozygous deletion of these tumor suppressors, both progeny cells and recruited cells expressed PDGFRα, with <5% tumors co-expressing EGFR (**Figure 4e**; **Figure S6a–c**; **data not shown**) [14]–[16], [33]. By contrast, glioma regions primarily comprised of eGFP-negative recruited cells frequently expressed EGFR or IGFR and not PDGFR (**Figure 4a–e** **; Figure S6b–d**). The primary mechanism for EGFR and IGFR expression in recruited regions is unclear; however, low-level amplification of these genes was seen in some cases. FISH analysis for mouse *EGFR* of eleven murine gliomas containing large regions of recruited cells with high expression of *EGFR* and/or *IGFR* showed >2 copies of *EGFR* in 5 of 11 cases, while 1 of 11 showed >2 copies of *IGFR* (**Figure S6e,f**). qPCR analysis of a panel of these and 15 other murine gliomas containing regions comprised of recruited cells was consistent with amplification of *EGFR* at the DNA level occurring at most in 6 of 18 (∼30%) of all cases, while *IGFR* was only amplified at most in 2 of 19 (∼10.5%) (**Figure S6g,h**).

In hPDGFb-induced gliomas, pharmacologic blockade by the PDGFR/VEGFR inhibitor PTK787 that crosses the blood-brain barrier results in cell cycle arrest of the tumor cells; thus, these gliomas retain their dependence on PDGFR signaling [39]. Since gliomas with large areas of recruited cells did not express PDGFRα, we investigated whether recruited cells became independent of hPDGFb signaling during glioma progression. We treated ten *Ntv-a Ink4a/Arf* ^+/-^ *Pten* ^+/fl^ glioma-bearing mice with PTK787 for a week at the onset of symptoms; eight mice survived the full course of treatment. Of these, three mice had gliomas that expressed PDGFRα and eGFP in high-grade tumor areas, concomitant with reduced proliferation rates (p<0.0001) (**Figure 4f**). Gliomas in the remaining five mice contained >85% recruited cells in high-grade tumor areas, showing proliferation rates statistically similar to the untreated tumors with large areas of recruitment (**Figure 4f** **; data not shown**). Thus, regions dominated by recruited cells became independent of PDGFR signaling during glioma progression, and the response to PTK787 treatment was dependent on the cellular composition of a given glioma. Although PTK787 also targets VEGFR, the conclusion that recruited glioma regions are independent of PDGFR activity is not confounded by this fact, since our readout is the relative lack of cell cycle arrest in the recruited glioma regions.

### Recruited cells initiate gliomas upon transplantation and aid glioma formation by progeny cells

**Figure 5:**
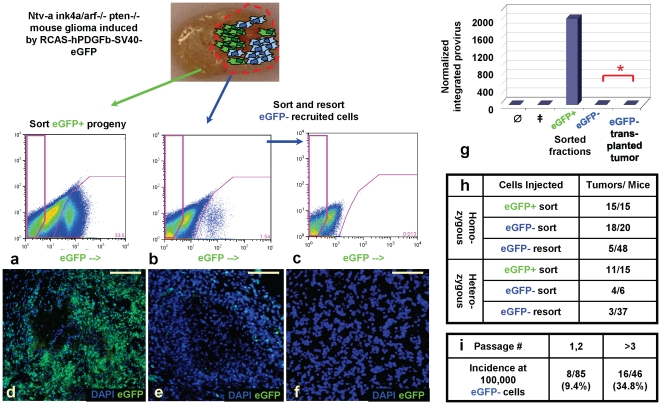
Recruited cells can form tumors upon transplantation and can be serially passaged in mice. FACS sorting of progeny (**a**) and FACS purification of the recruited cells (**b,c**) from RCAS-PSG-induced Ntv-a gliomas initiated in mice homozygous or heterozygous targeted for *Ink4a*, *Arf*, *Pten*. (**d–f**), DAPI-stained frozen sections showing virally encoded eGFP of transplanted gliomas induced by progeny (**d**), single-sorted recruited (**e**), or double-sorted recruited (**f**) cell fractions. (**g**) shows real-time qPCR analysis of sorted fractions and a transplanted eGFP-negative glioma induced by resorted recruited glioma cells. ∅, uninjected adult normal brain; ‡, Ras-driven Ntv-a glioma. All reactions were normalized to GAPDH. Graph shows fold change over the background amplification in Ras-driven glioma. (**h**) Incidence of gliomas induced by transplanting eGFP-positive progeny or double-purified eGFP-negative recruited cell fractions derived from *Ntv-a* RCAS-PSG-induced mouse gliomas homozygous or heterozygous deleted for *Ink4a*, *Arf* and *Pten* at tumor initiation. (**i**) Incidence of gliomas induced by serial transplantation of the recruited cells. Recruited cells can be serially passaged in mouse recipients, with a marked increase in frequency of tumor initiation after passage 3.

Although glioma patients do not die from glioma cells transplanted into them but rather from the massive expansion/infiltration by the glioma cells, tumor cells in rodent systems are most stringently defined by their ability to form secondary tumors upon transplantation into recipient mice [40**]**–[****44]. We isolated either progeny or recruited cells based on their expression of viral eGFP by FACS from *Ntv-a Ink4a/Arf* ^-/-^ *Pten* ^fl/fl^ or *Ink4a/Arf* ^+/-^ *Pten* ^+/fl^ mice injected with RCAS-PSG and RCAS-Cre (**Figure 5a,b** **; Figure S7e–h**). Single sorting of recruited cells (≥98.5% purity) gave rise to cell fractions that formed tumors with latency, grade and histology similar to gliomas induced by eGFP-positive progeny, but showed variable contamination with progeny cells in the resulting lesions with up to 10% progeny cells (**Figure 5d,e** **; Figure S7a–d**). Double sorting of eGFP-negative cells gave a population that was ≥99.5% recruited and formed high-grade gliomas not contaminated with progeny cells upon transplantation, albeit at a low frequency and requiring large numbers of transplanted cells (**Figure 5c, 5f–h** **; Figure S7e–h**). While large numbers of recruited cells were necessary to induce gliomas upon transplantation, it is important to remember that non-eGFP-expressing cells in virally-induced gliomas include normal stromal cells types (endothelial cells, astrocytes, macrophages, etc). Transplanted gliomas arising from stringently gated double-sorted eGFP-negative recruited cells did not express viral eGFP and had no detectable provirus by real-time PCR (**Figure 5g**). Moreover, these gliomas could be serially passaged (**Figure 5i**; **Figure S7i–l; Table S1**). In addition to EGFR expression and unlike gliomas with the typical oligodendroglial morphology induced by transplantation of progeny cells, these tumors showed regionally high expression of nestin, GFAP, CD44, vimentin and VEGFR, while expression of olig2 was variable in some tumor areas and absent in most (**Figure S8**) [**8**]. Thus, the recruited cells could form gliomas upon transplantation in some circumstances.

**Figure 6:**
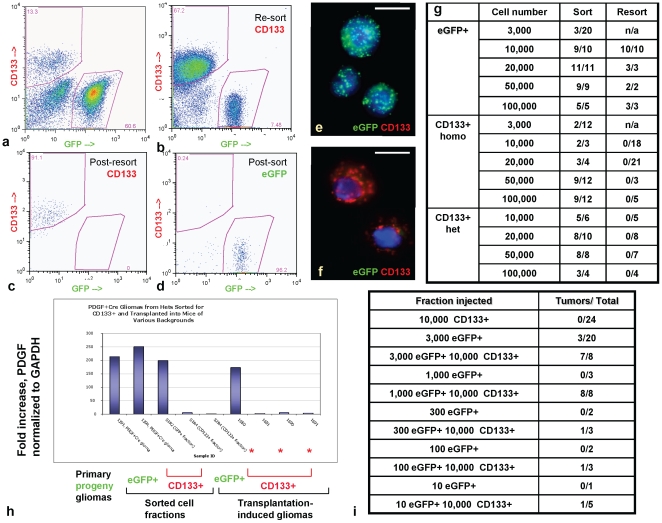
In RCAS-PSG-induced *Ntv-a* gliomas, CD133-positive cells are recruited and do not form gliomas upon transplantation, but contribute to gliomagenesis if co-transplanted with progeny. FACS purification and post-sort analysis of progeny (**a,d**) and FACS purification of the recruited CD133-positive cells (**b,c**) from RCAS-PSG-induced *Ntv-a* gliomas initiated in mice homozygous or heterozygous targeted for *Ink4a/Arf* and *Pten*. (**e,f**) FISH analysis for viral integration on the purified eGFP-positive progeny or CD133-positive recruited cells. Red background in (**f**) represents the CD133 staining. (**g**) Incidence of gliomas induced by transplanting eGFP-positive progeny or eGFP-negative CD133-positive recruited cell fractions derived from Ntv-a RCAS-PSG-induced mouse gliomas homozygous or heterozygous deleted for *Ink4a*, *Arf* and *Pten* at tumor initiation. (**h**) real-time qPCR analysis of sorted eGFP-positive cells, single-sorted CD133-positive cells and transplanted gliomas induced by these cell fractions, derived from Ntv-a gliomas initiated in mice heterozygous targeted for *Ink4a*, *Arf* and *Pten*. Asterisks indicate that transplanted gliomas induced by single-purified CD133-positive cells do not expand the progeny cell fraction. (**i**) Incidence of transplanted gliomas induced by injection of double-purified CD133-positive recruited cells, with or without low numbers of eGFP-positive progeny cells. Neither double-purified CD133-positive cells derived from RCAS-PSG-induced Ntv-a gliomas initiated in mice homozygous targeted for *Ink4a/Arf* and *Pten* at glioma initiation nor low numbers of eGFP-positive progeny give rise to gliomas on their own; however, addition of low numbers of recruited CD133-positive cells to few progeny cells allows for robust gliomagenesis.

CD133, a pentaspan transmembrane protein and a hematopoietic stem cell marker, was reported to label the most proliferative and robustly tumorigenic cells in human gliomas, although this has been questioned in different contexts [40], [42], [44]. We addressed tumorigenic properties of CD133-positive cells in the context of virally induced hPDGFb gliomas. Purified double-sorted CD133-positive cells derived from *Ntv-a Ink4a/Arf^-/-^Pten^fl/fl^* or *Ntv-a Ink4a/Arf^+/-^Pten^+/fl^* mice injected with RCAS-PSG and RCAS-Cre were not derived from progeny cells and were all recruited, as confirmed by RT-PCR, qPCR and FISH on FACS-sorted cells (**Figure 6a–f,h** **; Figure S9a–d**). Moreover, while transplantation of single-sorted CD133-positive cells resulted in gliomas contaminated by the eGFP-positive progeny, without the expansion of progeny cells in some cases (**Figure S9e–j;** **Figure 6h**), transplantation of purified CD133-positive recruited eGFP-negative cells from *Ntv-a Ink4a/Arf^-/-^Pten^fl/fl^* or *Ntv-a Ink4a/Arf^+/-^Pten^+/fl^* mice did not result in glioma formation, suggesting that in hPDGFb-driven murine gliomas, tumorigenicity lies in the CD133-negative recruited cell subset (**Figure 6g**).

We further addressed whether the CD133-positive subset of recruited cells showed functional importance for initiation or progression of transplanted hPDGFb-induced murine gliomas by cell-mixing experiments. While purified CD133-positive recruited cells could not give rise to transplanted gliomas at 10,000 cells (0/24 cases), and progeny cells did not robustly form tumors with cell numbers less than 3,000 (3/20 cases), addition of 10,000 CD133-positive recruited cells to 1,000 eGFP-positive progeny resulted in robust gliomagenesis (**Figure 6i**). Thus, CD133-positive recruited cells enhanced the ability of the progeny cells to initiate tumors upon transplantation, with small numbers of CD133-positive recruited cells sufficient to promote induction of transplanted tumors, the final mass of which was predominantly derived from the recruited cells (**data not shown**).

### Recruited cell gene expression resembles tumor cells

**Figure 7:**
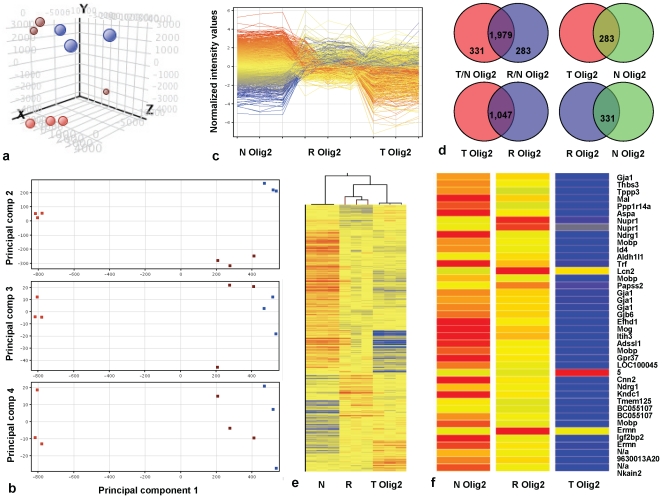
Gene expression of recruited olig2 cells resembles gene expression of olig2 glioma cells rather than adult olig2 progenitors. (**a,b**) Principal component analysis on filtered (**a**) and ANOVA-tested mRNAs (**b**) derived from normal olig2 progenitor cells (orange), glioma olig2 cells (blue) or recruited olig2 cells (brown). (**c**) Profile plot of non-averaged normalized intensity values of 2,593 ANOVA-tested mRNAs different between normal, glioma and recruited olig2 cell populations. Most mRNAs change in the same direction in recruited and tumor olig2 cells. (**d**) Venn diagrams showing differentially recruited polysomal mRNAs in glioma olig2 cells or recruited olig2 cells versus cortical olig2 progenitors (Diagram1), or polysomal mRNAs that are the same (Diagrams2,3,4) between indicated olig2 populations. T Olig2, tumor olig2 cells; R Olig2, recruited olig2 cells; N Olig2, normal adult cortical olig2-expressing progenitors. (**e**) Unsupervised hierarchical clustering on entities and conditions for N Olig2, T Olig2 or R Olig2 cells. Samples cluster within groups, and recruited olig2 cells cluster with tumor olig2 cells. (**f**) Heat map showing ANOVA-tested mRNAs over 10-fold enriched between recruited and glioma olig2 cell populations, as compared to normal olig2 cells.

An unbiased way to define a cell population lies in identification of its gene expression signature and subsequent comparison to gene expression signatures of known normal or cancer cells to define its position on the axis of tumorigenesis. To quantify similarities and differences in the expression profiles of recruited cells and tumor cells using microarray analysis, we used the *bacTRAP* technology that allows immunoprecipitation of polysomes from specific cell types *in vivo* (**Figure S3a–d**) [31], [32]. We compared polysomal expression profiles of hPDGFb-driven *Ntv-a Ink4a/Arf* ^+/-^ olig2 mouse glioma cells representing a histologically defined glioma population, recruited olig2 cells derived from *Ink4a/Arf* ^+/-^ *olig2 RP-eGFP* mice transplanted with non-fluorescent glioma cells, and normal adult cortical olig2 mouse progenitors, using Affymetrix 430 mouse 2.0 chips and Genespring GX10 software [31], [32]. Translational profiles of recruited olig2 cells and glioma olig2 cells were similar to each other and clearly distinct from the normal olig2 progenitors, majority of the differences accounted for by the “statistically significantly changed” set of mRNAs (ANOVA, p<0.05), and sample clustering reduced upon removal of the ANOVA-tested mRNA set (**Figure 7a–d**; **Figure S10a–c,e**). Unsupervised hierarchical mRNA clustering likewise indicated that recruited olig2 cells clustered more closely to glioma olig2 cells (**Figure 7e**). The caveat is that olig2 expression in the adult normal brain of *bacTRAP olig2 RP-eGFP* mice labels both progenitors and mature oligodendrocytes, while recruited and tumor olig2 cells are almost entirely progenitors; therefore, some of the differences seen in the comparative analysis of normal, tumor and recruited olig2 cells may in part result from the shift in relative abundance of different progenitor and mature olig2 cell populations.

Pair-wise comparisons indicated that out of 2,593 mRNAs differentially present on the polysomes, 2,310 mRNAs were differentially present in glioma vs. normal olig2 cells and 2,262 mRNAs in recruited vs. normal olig2 cells (one-way ANOVA, p<0.05, SNK post-hoc, FDR correction); of note, 86% (1,979 mRNAs) of these differentially represented mRNAs were the same (**Figure 7d**). Ingenuity analysis of this polysomal set revealed mRNAs implicated in genetic disorders, neurological disease and cancer, regulating cell death, morphology and the cell cycle (**data not shown**). Of 500 mRNAs most different between glioma and normal olig2 cells, 490 mRNAs were changed in the same direction but smaller magnitude in recruited olig2 cells, 440 of which were at least two-fold different, and 183 were five-fold different or more (**Figure S10d**). From the above data, the expression profile of recruited cells closely resembled that of tumor cells, placing the two closely together in the continuum of change towards the “malignant state”, and further confirming that recruited olig2 expressing cells are tumor. While only ∼300 mRNAs were the same between normal and either glioma or recruited olig2 cells, the number of mRNAs that were the same between glioma and recruited olig2 cells was at least three-fold more, amounting to 1,047 (**Figure 7d**). Many of these mRNAs were classified into gene categories listed above by Ingenuity (**data not shown**).

Although tumor and recruited olig2 cells were predominantly similar, the number of differentially recruited mRNAs with over 2-fold change between tumor and recruited olig2 populations amounted to 665, and those with over 5-fold change amounted to 132. mRNAs specifically present on polysomes of olig2 recruited cells coded for proteins in Hedgehog, Wnt, Ras, Rho/Rac and PLC pathways. While PDGFRa and TGFβ mRNAs were lower in recruited olig2 cells than tumor cells, mRNAs coding for EGFR, TGFα, TNF, LIF and FGFR1/2 were higher in recruited cells; prominin1 (CD133) mRNA was 5.3 fold higher in the recruited than tumor olig2 cells. mRNA populations corresponding to genes defining oligodendrocytic character were reduced in olig2 recruited cells, while mRNAs associated with astroglial and endothelial characteristics were upregulated in the recruited population. In addition, 41 mRNAs were increased on recruited cell polysomes by 10-fold or more, as compared to the tumor olig2 cells and differentially from normal olig2 cells (**Figure 7f**). Many of these mRNAs were gradually reduced in transition from normal to recruited and to tumor states, correlating with loss of oligodendroglial character (Aspa, Mobp, Tfr, Mog), evasion of cell death (Mal, Ndrg1, Gjb6, Gpr37), maintenance of undifferentiated phenotype (Mal, Ndrg1, Efhd1, Kndc1, Ermn), cell-cell and cell-matrix interactions (Thbs3, Tppp3, Itih3), and cell signaling (Ppp1r14a, Gja1). Cnn2 mRNA was specifically reduced on the polysomes of recruited cells as compared to both normal and tumor olig2 cells, while Nupr1, Aldh1l1, Lcn2, Papss2, and Igf2bp2 mRNAs were specifically increased in this population (**Figure 7f**).

## Discussion

RCAS/tv-a system allows to closely model the biology and histopathology of human oligodendrogliomas; although we have not performed direct comparisons between our mouse model and human glioma classifications, in many ways, hPDGFb-driven murine gliomas may mimic the proneural subclass of human GBMs [6]–[8]. Our data suggests that olig2-expressing cells recruited in PDGF-induced murine gliomas can have similar morphologic, proliferative and functional characteristics as olig2-expressing tumor cells derived from the cell-of-origin, with the caveat that a part of the similarity between the recruited and tumor olig2 cells may stem from their proliferative behavior. Extensive similarities between polysome-associated transcriptome of recruited versus glioma olig2 cells call for further evaluation of the precise definitions and criteria applied to terms “tumor” and “normal”, and indicate similar gene expression character of these cells. Furthermore, tumor suppressor loss and acquisition of mutations typically found in human gliomas allow these murine recruited cells to occupy large areas of the tumor and at times become the predominant cellular component of the glioma mass, completely independent of the cell-of-origin. Thus, the clonal expansion that overtakes glioma bulk during tumor progression need not be derived from glioma cell-of-origin.

Transformation is the process of self-autonomous acquisition of the sufficient oncogenic alterations that change a normal cell into the tumor cell. Unlike epithelial cancers, the “normal” or stromal component of a glioma is composed of cells of the same lineage as the tumor cells. The inherent implication of this observation is that extracellular factors that promote glioma tumor cell growth may similarly affect glioma stroma. In our glioma model, initially normal recruited cells not derived from the glioma-initiating cell-of-origin can be stimulated by hyperproduction of a growth factor receptor ligand, induced to proliferate, and driven to acquire various genetic aberrations and aberrant expression profiles as gliomas progress, presumably because of the tumor microenvironment created by the progeny of the cell-of-origin. The process of acquisition and selection for such genetic and/or epigenetic alterations due to the pressures of the glioma microenvironment may be better termed “corruption”. Although it is not known how frequently recruitment and subsequent corruption occurs in human PDGF-driven gliomas, gliomas in general or in other tumor types, our data indicates that this process may not be limited to hPDGFb-induced murine gliomas. As such, human gliomas that appear to be comprised of two or more genetically unrelated clones, or recurrent patient gliomas that lack the genetic alterations present in the originally resected tumors subjected to therapeutic stress, may be examples of these processes. Although similar processes have not been described *in vivo*, an *in vitro* line of evidence from the colon cancer cell lines suggested that one of the mechanisms of how cancer cells bypass senescence may be related to their potential for continuous “clonal diversification”, even within the what is perceived a clonal population of cancer cells, not affected by native stromal environment [45].

The process of corruption would thus occur if the tumor microenvironment initially drive proliferation and induce the stem-like character in both progeny of the cell-of-origin and the recruited cells. In hPDGFb-driven gliomas there is evidence for both - tumor cells secrete proliferative growth factors, inflammatory cells secrete cytokines, and endothelial and inflammatory cells secrete nitric oxide that activates the Notch signaling pathway, promoting the stem-like character [46]. Such environment affects cells within the tumor regardless of whether they are derived from the glioma-initiating cell-of-origin or not. This notion is further supported by the analysis of expression array data using the *bacTRAP olig2 RP-eGFP* reporter system and immunostaining, which suggest that recruited olig2 cells can acquire expression of GFAP and vimentin and increase the expression of nestin as compared to the tumor olig2 cells, triple co-expression of which is considered to be typical for the *bona fide* neural stem cells.

## Methods

### Ethics Statement

All animal experiments were conducted using protocols approved by the Institutional Animal Care and Use Committees of Memorial Sloan-Kettering Cancer Center. The approved protocols are: 00-11-189(MSKCC, last approved 3/15/2010).

#### Vectors, cell culture and Ntv-a tumor induction

RCAS-hPDGFb-HA-SV40-eGFP (RCAS-PSG) containing human PDGFb with partially deleted 5′UTR, RCAS-hPDGFb-HA, RCAS-Cre and RCAS-mRFP-SV40-Ras (RCAS-RSR) vectors were transfected into DF-1 chicken cells (ATCC) [14], [37]. DF1 cells transfected with RCAS-PSG were FACS-sorted to select 15% brightest eGFP-expressing cells and cultured in 10% FBS-DMEM media under standard conditions. For tumor induction, transfected DF1 cells were trypsinized, centrifuged and injected into the brain parenchyma of Ntv-a mice that transgenically express RCAS-binding tv-a receptor after the nestin promoter, at post-natal days 0–2. At 3 weeks post-injection, injected Ntv-a mice were weaned and observed daily for the appearance of brain tumor symptoms.

#### Tissue collection and processing

Experiments involving mice were performed in accordance with the regulations set by the MSKCC IACUC committee. Upon the appearance of brain tumor symptoms (e.g. poor grooming, lethargy, macrocephaly, hydrocephaly, hemiparesis, weight loss), mice were anesthetized by an intraperitoneal injection of 1 mg/kg Nembutal solution or ketamine (150 mg/kg)/xylazine (15 mg/kg) cocktail and underwent trans-cardiac perfusion with 10 ml ice-cold heparin normal saline, followed by 10 ml of ice-cold 4% paraformaldehyde. Brain tissue was extracted and post-fixed for 30 minutes in ice-cold 4% paraformaldehyde, transferred into 30% sucrose at 4°C for cryoprotection, embedded using the OCT compound, frozen on dry ice and stored at −80°C until sectioned. A small subset of animals were sacrificed using CO_2_, their brain tissue extracted and fixed for 3 days in 4% paraformaldehyde at 4°C, and subsequently processed using standard paraffin embedding and processing techniques.

#### bacTRAP technology

Normal olig2 progenitors were collected from three replicates of pooled cortices of three *olig2 RP-eGFP bacTRAP* reporter mice. Mouse gliomas induced by injection of a non-fluorescent RCAS-hPDGFb [14] or by transplantation of murine glioma cells derived from Ntv-a gliomas induced by the non-fluorescent RCAS-hPDGFb were collected from *Ntv-a olig2 RP-eGFP bacTRAP* reporter mice and immunoprecipitated; each tumor was processed as a separate sample. Briefly, mouse tissues were collected into ice-cold cyclohexamide-containing buffer, homogenized, cells were lysed in NP-40 and DHPC-containing buffer, centrifuged at 20,000 g for 15 min, supernatant incubated with anti-eGFP-conjugated protein G beads 30 min at 4C, washed and RNA collected using Trizol reagent as per manufacturer's instructions [31], [32]. RNA was purified, concentrated using Qiagen RNeasy kit, its quality confirmed by Agilent Bioanalyzer, 15 ng per sample amplified using Affymetrix Two-Cycle Amplification kit and hybridized to Affymetrix 430 mouse 2.0 chips as described in [31], [32].

#### Microarray analysis

Data was analyzed using Genespring GX10 software, as described in the text and in [31], [32]. Briefly, samples were normalized using GCRMA, filtered to remove probe sets with low intensity, and analyzed using ANOVA (p<0.05) with SNK post-hoc and Benjamini and Hochberg FDR multiple testing correction. Probe quality, Pearson's correlation, hierarchical clustering, heatmaps, gene profile plots were generated using Genespring GX10 software. Pathway analysis was performed using Ingenuity as per manufacturer's instructions. The mRNA microarray data generated by this study are available through the NCBI Gene Expression Omnibus (GEO) (accession number GSE30016, http://www.ncbi.nlm.nih.gov/geo/query/acc.cgi?acc=GSE30016).

#### Cell Sorting and transplantation of murine glioma cells

Glioma-bearing brains were extracted from anesthetized mice, gliomas excised from surrounding brain parenchyma, dissociated using a mix of collagenases as previously described, and sorted using MoFlo FACS sorting. Various numbers of cells (1,000–500,000) resuspended in sterile 1X PBS were injected into brain parenchyma of neonatal immunonaive mice of various genetic backgrounds, or stereotactically transplanted into anesthetized adult SCID mice.

#### PCR on tumor sections

Mouse glioma sections were scraped from slides and lyzed with 0.05% SDS buffer. Genomic DNA was extracted using phenol-chloroform according to standard techniques, and precipitated at −20°C overnight. Prior to PCR for hPDGFb, DNA was heated at 56°C for 2 hours or for 5 min at 100°C, concentration measured, and equal amounts of DNA added to PCR reactions. PCR reactions for human PDGFB, *Pten*, and the *Ink4a/Arf* locus were performed using previously published primers. Control GAPDH reactions were performed in the same PCR mix. Data was obtained using GelDoc Software.

#### qPCR on tumor sections

DNA was extracted using 0.05% SDS buffer as above; Roche primers were used to detect amplifications of mouse *EGFR* (Set#6, AGAACCAGTCCAGATCGG and AAGGATGTGTAAGGGACC; Set#27, ACACTTGCCCAGAAAAATG and AGGTGGGATTGTACAGAG) or mouse *IGFR* (Set#67, CTCACAGGTGGACAGTGC and ATGCCCCAGGGCCTGTAA; Set#104, GAGAATTTCCTTCACAATT and CACTTGCATGACGTCTCTC). *Pten* qPCR was performed using previously published primers [37].

#### RT-PCR on FACS-sorted cells

Total RNA from sorted cells was extracted using the Qiagen RNeasy kit and resuspended in 30-50 ul of DEPC-treated water. About 5 ug of total RNA was reverse-transcribed using the SuperScriptIII Reverse strand synthesis kit from Invitrogen. Quantitative RT-PCR was performed using primers for human PDGFb and mouse actin as a control.

#### Immunohistochemistry

Cryogenically processed murine glioma tissue was sectioned at 7 microns, washed with PBS, blocked with 10% serum in PBS using 0–0.3% TX-100 for polyclonal rabbit/goat or MOM kit for monoclonal mouse and rat antibodies, and incubated with primary antibodies for 1 hour at room temperature or overnight at 4C. The primary antibodies included PDGFRα and PTEN from Cell Signaling; olig2, Ki67, hNA and EGFR from Abcam; NeuN from Chemicon; HA from Santa Cruz; and eGFP from Molecular Probes. Secondary fluorochrome-conjugated antibodies (Alexa555, Alexa488, and Alexa647) were applied for 1 hr at room temperature, slides washed several times, counterstained with DAPI (1∶3000 of a 1 mg/ml 1X PBS solution), mounted in 70% glycerol, coverslipped and photographed with inverted fluorescent or confocal Leica microscopes.

#### Cell Sorting and transplantation of murine glioma cells

Glioma-bearing brains were extracted from anesthetized mice, gliomas excised from surrounding brain parenchyma, dissociated using a mix of collagenases as previously described, and sorted using DAKO-Cytomation MoFlo FACS sorters for eGFP expression. Samples were gated to exclude debree, dead cells and doublets. Various numbers of cells (1,000–500,000) resuspended in sterile 1X PBS were injected into the brain parenchyma of neonatal immunonaive mice of various Ntv-a genetic backgrounds, or stereotactically transplanted into anesthetized adult SCID mice, using Hamilton syringe. CD133 (eBioscience) staining of acutely dissociated murine glioma cells was done as per manufacturer's instructions; CD133 antibody was used at a 10-fold lower concentration based on titration. Single- or double-sorted CD133-positive cells were transplanted as described above. Cell-mixing experiments were performed using double-sorted eGFP-positive progeny and double-sorted CD133-positive recruited cells, which were mixed just prior to transplantation, and injected using Hamilton syringe.

#### Fluorescent in-situ hybridization

RCAS DNA was fragmented using nick translation kit, labeled with biotin, and digested to 500 bp-2 kb probe size. Resulting probes were denatured at 80°C and applied to tumor sections blocked with salmon sperm DNA for an overnight incubation at 37°C. Slides were washed with 2X SSC-50% formamide solution at 45°C, signal amplified according to standard techniques using TSA kit, and revealed with Alexa488 and Alexa555. Slides were counterstained with DAPI (1∶3000 of 1 mg/ml PBS solution), mounted, coverslipped and photographed using inverted fluorescent or confocal Leica microscopes.

#### Statistical analysis

Kaplan-Meyer curves for mouse glioma latency were made using Prism4 software and analyzed with standard log rank test, or using R. The p value was determined to be less than 0.001 for survival curves of *Ntv-a* wild-type vs. *Ntv-a Arf^-/-^* mice injected with RCAS-PSG vs. *Ntv-a Ink4a/Arf^-/-^ Pten^fl/fl^* mice injected with RCAS-PSG and RCAS-Cre. Survival curves for *Ntv-a* wild-type vs. *Ntv-a Ink4a^-/-^*, or for *Ntv-a Ink4a/Arf^-/-^* vs. *Ntv-a Arf^-/-^*, were not statistically different from each other. Statistical analysis of other data, including percentages of eGFP-positive cells in different murine Ntv-a backgrounds and percentages of Ki67-positive cells in PTK787-treated murine gliomas, was performed using unpaired two-tailed t-tests and F-tests in Prism4.

## Supporting Information

Figure S1 **Background information for RCAS-PSG-induced** ***Ntv-a*** **gliomas.** (**a–d**) H&E-stained paraffin sections of low-grade Ntv-a gliomas containing secondary structures of Scherer characteristic of human gliomas: subpial accumulations of tumor cells (**a**), white matter tracking (**b**), perivascular (**c**) and perineuronal satellitosis (**d**). Red arrowheads indicate glioma cells. (**e,f**) Expression of eGFP in high-grade *Ntv-a Arf^-/-^* murine gliomas is detectable by FACS (**e**), and correlates with RCAS-hPDGFb infection. eGFP+ and eGFP- glioma cells were sorted and used for real-time PCR. (*Ntv-a Arf^-/-^* high-grade gliomas were used in lieu of wild-type because wild-type Ntv-a gliomas contained very few recruited cells.) (**f**) shows normal murine *Ntv-a* brain, total mixed tumor cell population, and sorted progeny or recruited cell fractions for *Ntv-a* gliomas shown in (**e**).
(TIF)

Figure S2 **Expression of eGFP in progeny cells of low- and high-grade** ***Ntv-a*** **gliomas correlates with hPDGFb expression.** Images (**a–d**) show native eGFP, anti-HA staining for the hemagglutinin tag on viral hPDGFb and corresponding composites in low-grade (**a**,**b**) and high-grade (**c,d**) Ntv-a gliomas. Overall expression of eGFP and hPDGFb correlates on a cell-to-cell basis, but relative amounts of expression of eGFP and hPDGFb proteins may differ. (**e**,**f**) High-magnification composites showing correlation of hPDGFb and eGFP expression in pseudopalisade regions of high-grade Ntv-a gliomas. Asterisks in (**e**,**f**) indicate pseudopalisade lumens.
(TIF)

Figure S3 **Validation of the** ***bacTRAP olig2 RP-eGFP*** **system in** ***Ntv-a*** **gliomas.** (**a**) olig2 (red) and nestin (green) immunostaining of *Ntv-a* wild-type mouse RCAS-hPDGFb-induced gliomas, showing expression of olig2 in most tumor cells. (**b**) Experimental design to label and isolate olig2 tumor cells and olig2 recruited cells. (**c,d**) DAPI-stained frozen sections of murine gliomas induced in *bacTRAP olig2 RP-eGFP* reporter mice, with olig2 and eGFP stains shown in red. *bacTRAP olig2 RP-eGFP* mice accurately recapitulate expression of olig2 and eGFP.
(TIF)

Figure S4 **Tumor suppressor loss in hPDGFb-induced Ntv-a gliomas results in shorter glioma latency, increased grade and larger numbers of recruited cells.** (**a**) H&E-stained section of an *Ntv-a* glioma containing pseudopalisading necrosis and microvascular proliferation, red arrowheads. (**b**) Tumor incidence and presence of low- and high-grade glioma structures in gliomas of various *Ntv-a* mouse backgrounds, induced with RCAS-PSG with or without RCAS-Cre. X-axes color-coding corresponds to color-coding of *Ntv-a* mouse strains in the Kaplan-Meyer analysis in Figure 2. (**c**) Correlations between PTEN immunostaining (IHC) and *Pten* LOH (PCR for *Pten* alleles on tumor cell DNA extracted from mouse glioma sections) during glioma progression in *Ntv-a* gliomas heterozygous targeted for *Ink4a*, *Arf* and *Pten* tumor suppressor loss at glioma initiation. DNA concentrations were measured, and equal amounts of DNA were loaded per tumor sample. Normal, uninjected murine *Ntv-a* brain; PDGF and PDGF/Cre, murine gliomas induced in *Ntv-a* mice homozygous targeted for *Ink4a*, *Arf* and *Pten* loss at glioma initiation. All other samples are derived from *Ntv-a Ink4a/Arf^+/-^Pten^+/fl^* mice injected with RCAS-PSG and RCAS-Cre. Note lack of *Pten* expression in some murine gliomas that retain *Pten* at the genetic level. (**d**,**e**) PTEN expression is absent in Ntv-a GBMs heterozygous targeted for *Ink4a*, *Arf* and *Pten* tumor suppressor loss at glioma initiation.
(TIF)

Figure S5 **The recruitment phenomenon is not limited to murine gliomas characterized by paracrine hPDGFb signaling: K-Ras-driven** ***Ntv-a*** **gliomas with tumor suppressor loss contain recruited cells.** **Quantification of recruited cells and comparison of glioma latency in GBMs with large regions of recruitment or progeny cell contribution.** (**a**) Construction of RCAS-mRFP-SV40-KRasG12D vector. (**b,c**) mRFP expression DF1 cells transfected with RCAS-mRFP-SV40-KRasG12D. (**d,e**) Expression of mRFP in frozen sections of an *Ntv-a* GBMs induced by injection of RCAS-mRFP-SV40-KRasG12D and RCAS-Cre, mRFP immunostaining. Note the presence of cells not expressing mRFP. (**f**) Immunostaining for *RP-eGFP* expression (red) in recruited olig2 cells in transplanted gliomas arising in *bacTRAP olig2 RP-eGFP* mice induced by transplantation of Ras-driven Pten-deleted murine glioma cells. (**g**) PDGFRα stain (red) of an Ntv-a glioma induced by RCAS-K-RasG12D and RCAS-Cre. Note absence of PDGFRα expression in tumor cells. (**h**) *RP-eGFP* is expressed in olig2 tumor cells contributing to pseudopalisades of transplanted gliomas induced by the hPDGFb-expressing murine glioma cells and arising in *bacTRAP olig2 RP-eGFP* mice. (**i**) Tumor latency for *Ntv-a* gliomas containing large regions of progeny or recruited cells induced in *Ntv-a* mice heterozygous targeted for loss of Ink4a, Arf and Pten by RCAS-PSG and RCAS-Cre. While there is a small but statistically significant difference with respect to latency medians (two-tailed unpaired Student's t-test, p<0.006), latency variances largely overlap (F test). (**j**) FACS plots of 4 *Ntv-a* gliomas heterozygous targeted for tumor suppressor loss of *Ink4a*, *Arf* and *Pten* at glioma initiation, containing large numbers of recruited cell pseudopalisades. Percentages of recruited cells vary from 0.91% to 36.8%. (**k**) Graph shows variability of contribution from the eGFP-expressing progeny and the eGFP-negative recruited cells across various *Ntv-a* mouse strains, including *Ntv-a* gliomas containing large regions of recruitment.
(TIF)

Figure S6 **hPDGFb-induced** ***Ntv-a*** **gliomas show regional expression of growth factor receptors important in human glioma biology, expression of which in recruited cells may be associated with low level amplifications in some cases.** (**a–d**) *Ntv-a* gliomas induced in *Ntv-a Ink4a/Arf^+/-^Pten^+/fl^* mice injected with RCAS-PSG and RCAS-Cre stained with IGFR (red), EGFR (pink) (**a,d**) or PDGFRα (red) (**b,c**). Glioma regions predominantly derived from progeny cells (**a,b**) express PDGFRα; expression of EGFR and IGFR is limited to perivascular areas. (**c,d**) Adjacent sections stained with PDGFRα and EGFR (red) show regional expression of growth factor receptors; eGFP omitted for easier view. (**e**) FISH for IGFR (green) and EGFR (red) on Ntv-a GBMs with large areas of recruitment and high EGFR expression. Panels show a neuron, EGFR-expressing non-amplified GBM with extensive recruitment, and EGFR-expressing GBM with EGFR amplification in the recruited cells. (**f**) Summary of mEGFR FISH performed on EGFR-expressing GBMs with large areas of recruitment. Gliomas containing more than 15% of cells with ≥3 mEGFR signals are marked with an asterisk (amplified). (**g,h**) real-time qPCR on DNA extracted from sections of mouse Ntv-a GBMs containing large areas of recruitment (blue squares) highly expressing EGFR. Primers for mEGFR kinase domain (**g**) or intron region of mEGFR (**h**) were designed by Roche; graphs show fold increase over normal EGFR copy number (∅); gliomas with large areas composed of progeny cells (green squares) were used to establish range of variability of primer noise. Gliomas marked with asterisks were considered amplified, and gliomas marked with arrows appeared amplified by both primer pairs, potentially representing tumors with larger chromosomal amplifications.
(TIF)

Figure S7 **Recruited cells can initiate gliomas upon transplantation and can be serially passaged in mouse hosts.** (**a–d**) H&E-stained sections depicting histology of mouse gliomas induced by transplanting recruited cells. (**e–g**) Purification of recruited cells from *Ntv-a Ink4a/Arf^+/-^Pten^+/fl^* mice injected with RCAS-PSG and RCAS-Cre; (**h**) DAPI-stained frozen section of a glioma induced by recruited cells; note absence of cells with virally encoded eGFP. (**i–l**) Gliomas induced by the recruited cells could be serially passaged in mice. (**i–k**), FACS plots of gliomas induced by serial transplantation of recruited cells into mouse hosts at passaging; recruited glioma cells were single purified between passages. (**l**) DAPI-stained section of a transplanted glioma induced by recruited cells, showing absence of progeny cells with virally encoded eGFP.
(TIF)

Figure S8 **Immunohistochemical analysis of murine gliomas induced by transplanting recruited cells.** Images (**a–l**) show native eGFP and immunostaining for Ki-67 (**a**,**b**), olig2 (**c**,**d**), GFAP (**e**,**f**), nestin (**g**,**h**), VEGFR (**i**), YKL40 (**j**) and CD44 (**k**,**l**).
(TIF)

Figure S9 **Single-sorted CD133-positive cells are contaminated by low numbers of progeny cells and give rise to gliomas containing small numbers of progeny.** FACS plot of an Ntv-a GBM induced by RCAS-PSG and RCAS-Cre, stained with CD133. Single-purified (**b,c**) CD133-positive cells are recruited, but sorted fractions contain low numbers of progeny cells. Double-purified CD133 cells are recruited (**d**). (**b**) PCR and qPCR (**c,d**) analysis of sorted CD133-positive recruited cells and eGFP-positive progeny, showing absence of viral integration. (**e–j**) H&E and DAPI-stained sections of transplanted gliomas induced by single-sorted eGFP-positive (**e,h**) or CD133-positive (**f,g,i,j**) cells. Note the presence of contaminating progeny in transplanted gliomas induced by single-sorted CD133-positive cell fractions.
(TIF)

Figure S10 **Microarray analysis of normal olig2, recruited olig2 and glioma olig2 cells indicates that recruited cells are similar to glioma cells and differ from normal progenitors.** (**a**) Glioma induced by transplantation of non-fluorescent hPDGFb-driven mouse glioma cells into wild-type *olig2 RP-eGFP* bacTRAP reporters, showing transgenic *RP-eGFP* expression and anti-eGFP stain (red). Note large numbers of RP-eGFP-positive recruited cells. (**b,c**) Quality Controls Metrics plot and Pearson's correlation plot for Affymetrix arrays for normal (N), tumor (T) or recruited (R) olig2 cells. (**d**) Genespring GX10-generated profile plots showing averaged normalized intensity values for 500 mRNAs most different between glioma and normal olig2 cells (first plot), changing at least two-fold (second plot) or at least five-fold (third plot) between recruited olig2 cells and normal olig2 progenitor cells. (**e**) Principal component analysis on mRNAs with ANOVA-tested mRNA set removed. Note that all samples do not cluster well when the ANOVA-tested set of mRNAs specifically changed in tumor olig2 cells versus normal olig2 cells is removed.
(TIF)

Table S1 **The dilution table for transplanted double-sorted recruited eGFP-negative cells.** Incidence of gliomas induced by transplanting double-purified eGFP-negative recruited cell fractions derived from *Ntv-a* RCAS-PSG-induced mouse gliomas homozygous or heterozygous deleted for *Ink4a*, *Arf* and *Pten* at tumor initiation. Shown in the Table are numbers of large tumors and smaller proliferative lesions arising in recipient mice.
(TIF)
