# Supplementary figures and images for: Recruited Cells Can Become Transformed and Overtake PDGF-Induced Murine Gliomas In Vivo during Tumor Progression
Source: PLoS One. 2011 Jul 6;6(7):e20605. doi: 10.1371/journal.pone.0020605 (PMC3130733; doi:10.1371/journal.pone.0020605)

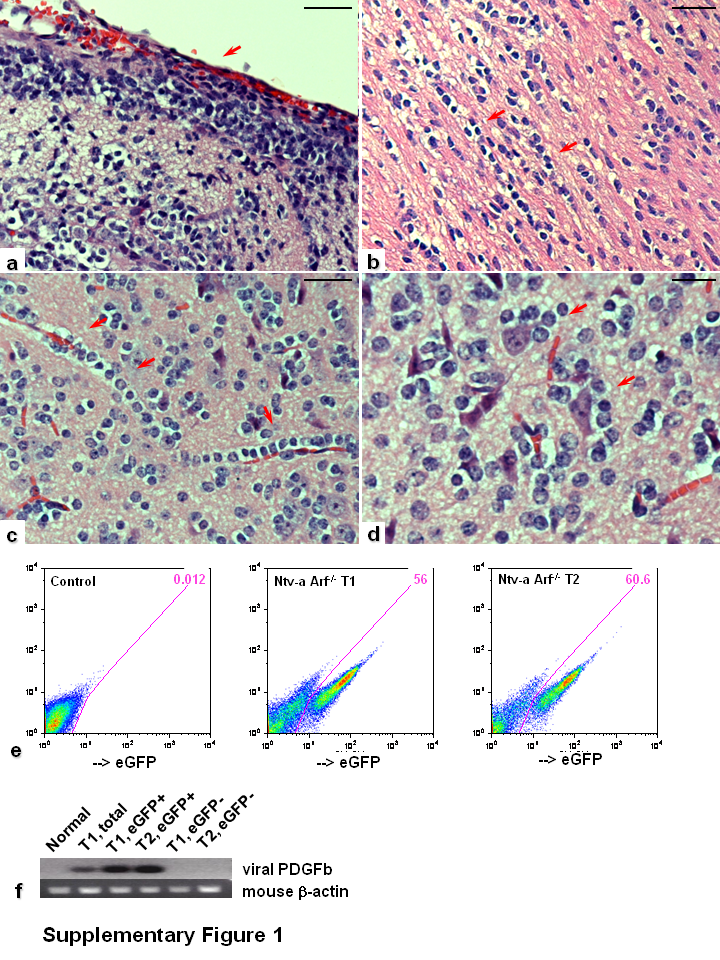

Supplement: Figure S1 — Background information for RCAS-PSG-induced Ntv-a gliomas. (a–d) H&E-stained paraffin sections of low-grade Ntv-a gliomas containing secondary structures of Scherer characteristic of human gliomas: subpial accumulations of tumor cells (a), white matter tracking (b), perivascular (c) and perineuronal satellitosis (d). Red arrowheads indicate glioma cells. (e,f) Expression of eGFP in high-grade Ntv-a Arf-/- murine gliomas is detectable by FACS (e), and correlates with RCAS-hPDGFb infection. eGFP+ and eGFP- glioma cells were sorted and used for real-time PCR. (Ntv-a Arf-/- high-grade gliomas were used in lieu of wild-type because wild-type Ntv-a gliomas contained very few recruited cells.) (f) shows normal murine Ntv-a brain, total mixed tumor cell population, and sorted progeny or recruited cell fractions for Ntv-a gliomas shown in (e). (TIF) [file pone.0020605.s001.tif]

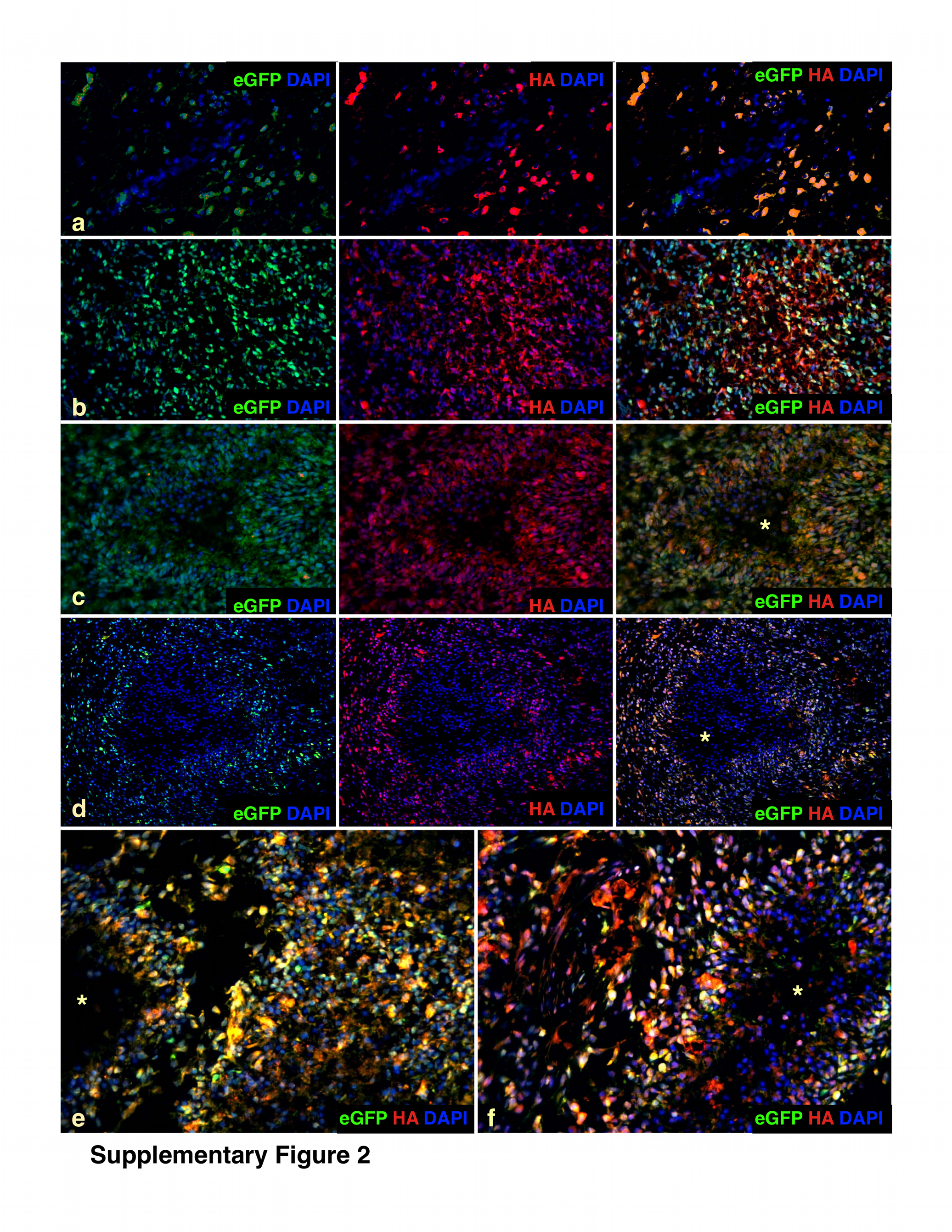

Supplement: Figure S2 — Expression of eGFP in progeny cells of low- and high-grade Ntv-a gliomas correlates with hPDGFb expression. Images (a–d) show native eGFP, anti-HA staining for the hemagglutinin tag on viral hPDGFb and corresponding composites in low-grade (a,b) and high-grade (c,d) Ntv-a gliomas. Overall expression of eGFP and hPDGFb correlates on a cell-to-cell basis, but relative amounts of expression of eGFP and hPDGFb proteins may differ. (e,f) High-magnification composites showing correlation of hPDGFb and eGFP expression in pseudopalisade regions of high-grade Ntv-a gliomas. Asterisks in (e,f) indicate pseudopalisade lumens. (TIF) [file pone.0020605.s002.tif]

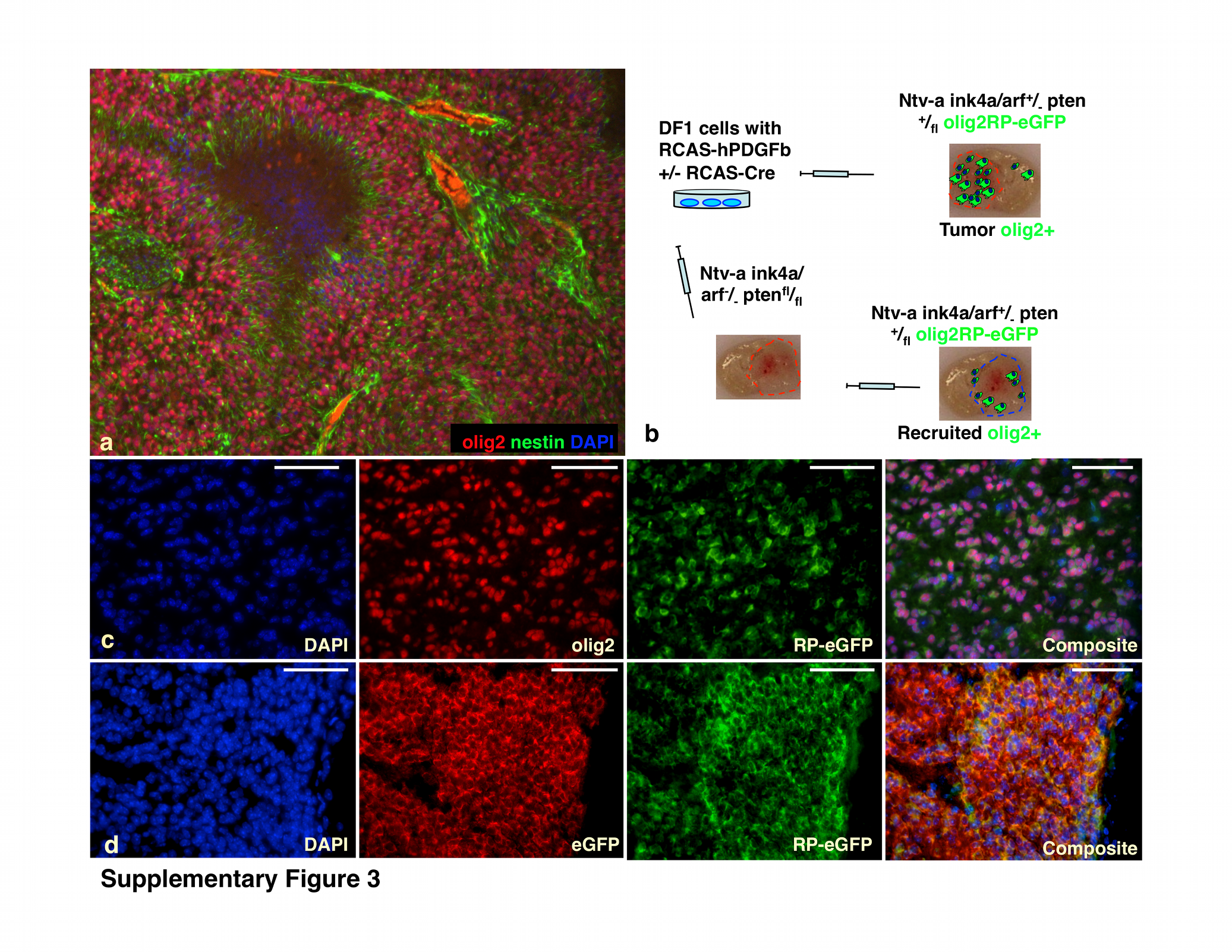

Supplement: Figure S3 — Validation of the bacTRAP olig2 RP-eGFP system in Ntv-a gliomas. (a) olig2 (red) and nestin (green) immunostaining of Ntv-a wild-type mouse RCAS-hPDGFb-induced gliomas, showing expression of olig2 in most tumor cells. (b) Experimental design to label and isolate olig2 tumor cells and olig2 recruited cells. (c,d) DAPI-stained frozen sections of murine gliomas induced in bacTRAP olig2 RP-eGFP reporter mice, with olig2 and eGFP stains shown in red. bacTRAP olig2 RP-eGFP mice accurately recapitulate expression of olig2 and eGFP. (TIF) [file pone.0020605.s003.tif]

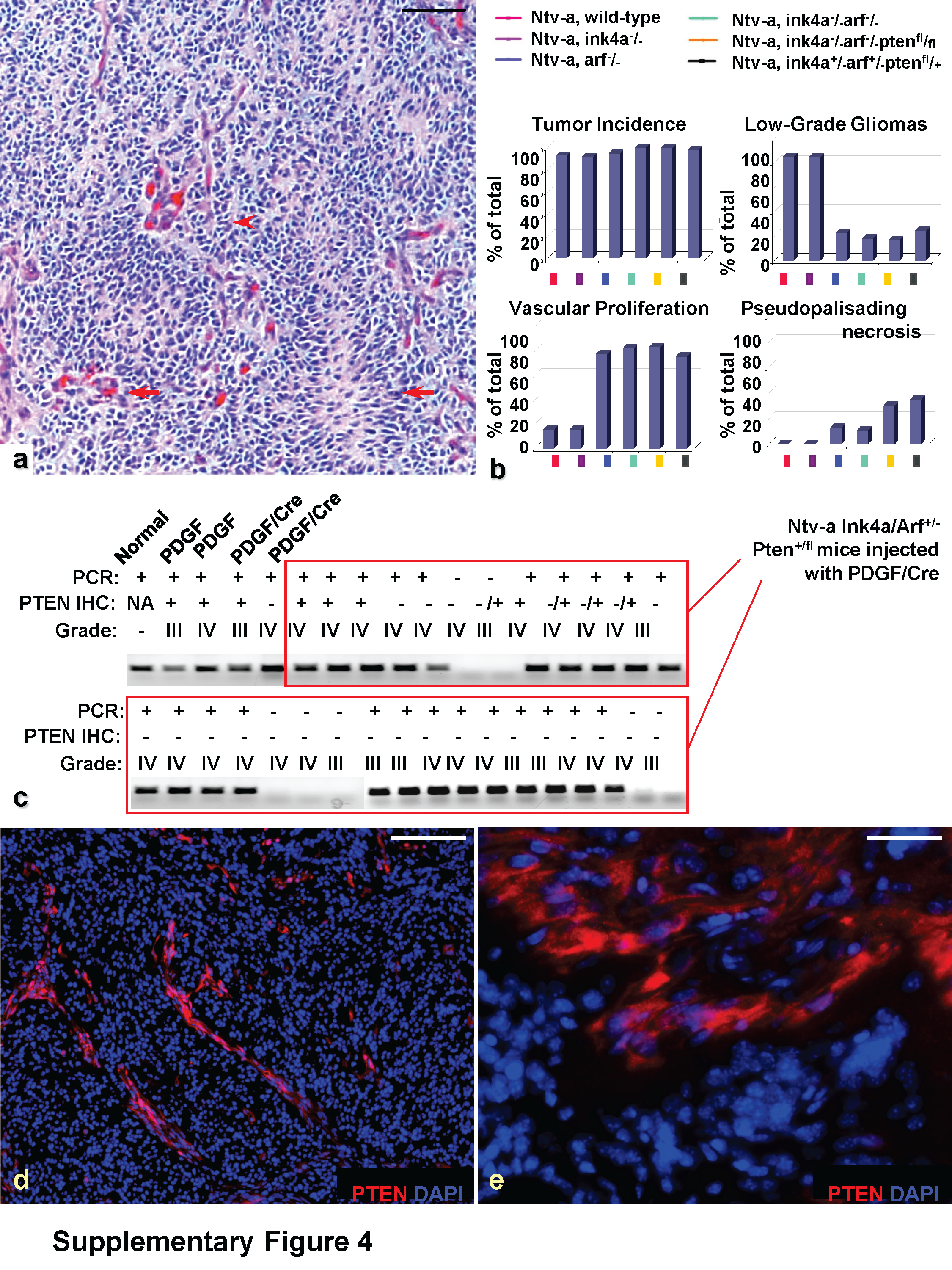

Supplement: Figure S4 — Tumor suppressor loss in hPDGFb-induced Ntv-a gliomas results in shorter glioma latency, increased grade and larger numbers of recruited cells. (a) H&E-stained section of an Ntv-a glioma containing pseudopalisading necrosis and microvascular proliferation, red arrowheads. (b) Tumor incidence and presence of low- and high-grade glioma structures in gliomas of various Ntv-a mouse backgrounds, induced with RCAS-PSG with or without RCAS-Cre. X-axes color-coding corresponds to color-coding of Ntv-a mouse strains in the Kaplan-Meyer analysis in Figure 2. (c) Correlations between PTEN immunostaining (IHC) and Pten LOH (PCR for Pten alleles on tumor cell DNA extracted from mouse glioma sections) during glioma progression in Ntv-a gliomas heterozygous targeted for Ink4a, Arf and Pten tumor suppressor loss at glioma initiation. DNA concentrations were measured, and equal amounts of DNA were loaded per tumor sample. Normal, uninjected murine Ntv-a brain; PDGF and PDGF/Cre, murine gliomas induced in Ntv-a mice homozygous targeted for Ink4a, Arf and Pten loss at glioma initiation. All other samples are derived from Ntv-a Ink4a/Arf+/-Pten+/fl mice injected with RCAS-PSG and RCAS-Cre. Note lack of Pten expression in some murine gliomas that retain Pten at the genetic level. (d,e) PTEN expression is absent in Ntv-a GBMs heterozygous targeted for Ink4a, Arf and Pten tumor suppressor loss at glioma initiation. (TIF) [file pone.0020605.s004.tif]

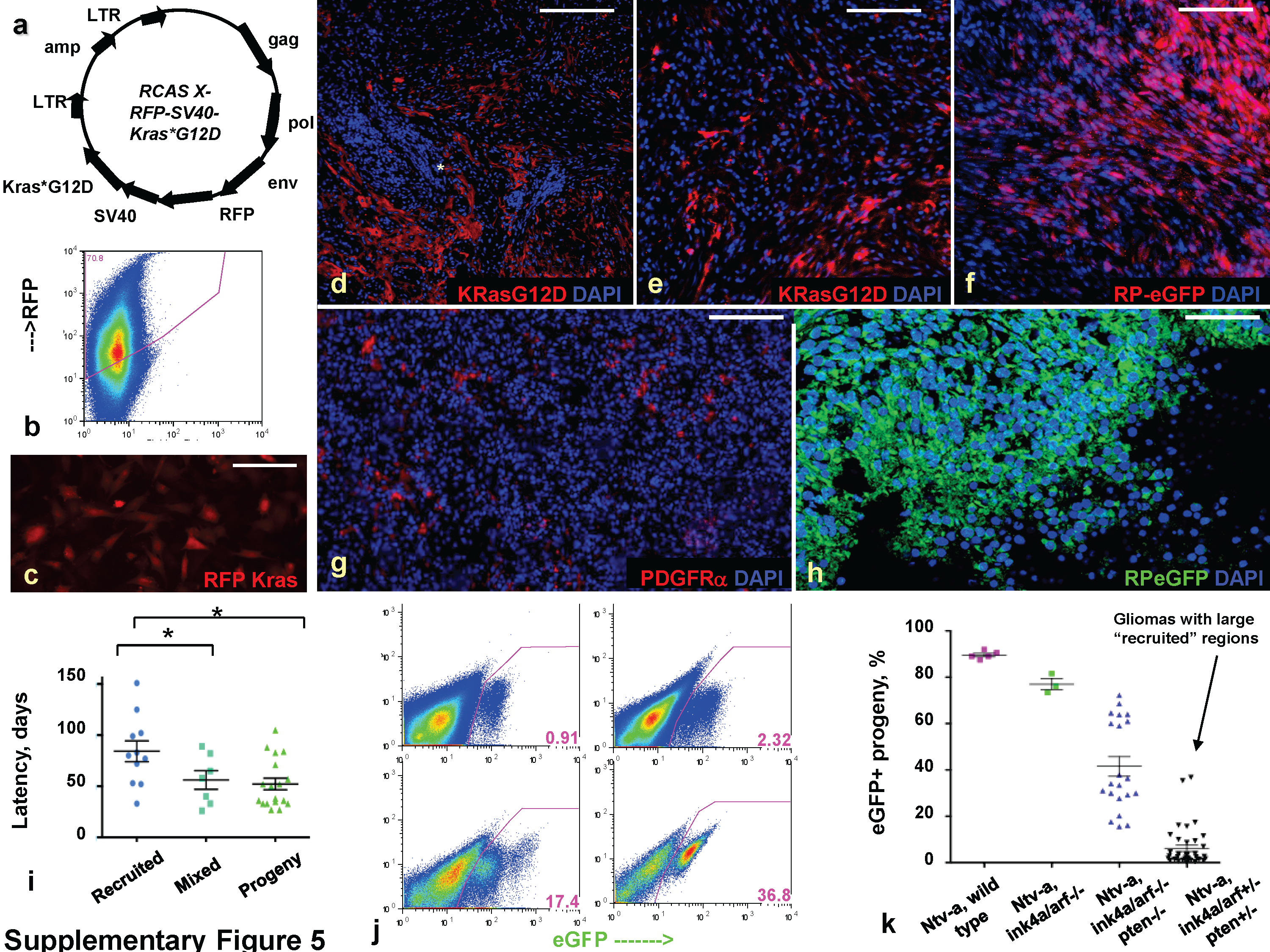

Supplement: Figure S5 — The recruitment phenomenon is not limited to murine gliomas characterized by paracrine hPDGFb signaling: K-Ras-driven Ntv-a gliomas with tumor suppressor loss contain recruited cells. Quantification of recruited cells and comparison of glioma latency in GBMs with large regions of recruitment or progeny cell contribution. (a) Construction of RCAS-mRFP-SV40-KRasG12D vector. (b,c) mRFP expression DF1 cells transfected with RCAS-mRFP-SV40-KRasG12D. (d,e) Expression of mRFP in frozen sections of an Ntv-a GBMs induced by injection of RCAS-mRFP-SV40-KRasG12D and RCAS-Cre, mRFP immunostaining. Note the presence of cells not expressing mRFP. (f) Immunostaining for RP-eGFP expression (red) in recruited olig2 cells in transplanted gliomas arising in bacTRAP olig2 RP-eGFP mice induced by transplantation of Ras-driven Pten-deleted murine glioma cells. (g) PDGFRα stain (red) of an Ntv-a glioma induced by RCAS-K-RasG12D and RCAS-Cre. Note absence of PDGFRα expression in tumor cells. (h) RP-eGFP is expressed in olig2 tumor cells contributing to pseudopalisades of transplanted gliomas induced by the hPDGFb-expressing murine glioma cells and arising in bacTRAP olig2 RP-eGFP mice. (i) Tumor latency for Ntv-a gliomas containing large regions of progeny or recruited cells induced in Ntv-a mice heterozygous targeted for loss of Ink4a, Arf and Pten by RCAS-PSG and RCAS-Cre. While there is a small but statistically significant difference with respect to latency medians (two-tailed unpaired Student's t-test, p<0.006), latency variances largely overlap (F test). (j) FACS plots of 4 Ntv-a gliomas heterozygous targeted for tumor suppressor loss of Ink4a, Arf and Pten at glioma initiation, containing large numbers of recruited cell pseudopalisades. Percentages of recruited cells vary from 0.91% to 36.8%. (k) Graph shows variability of contribution from the eGFP-expressing progeny and the eGFP-negative recruited cells across various Ntv-a mouse strains, including Ntv-a gliomas containing large r [file pone.0020605.s005.tif]

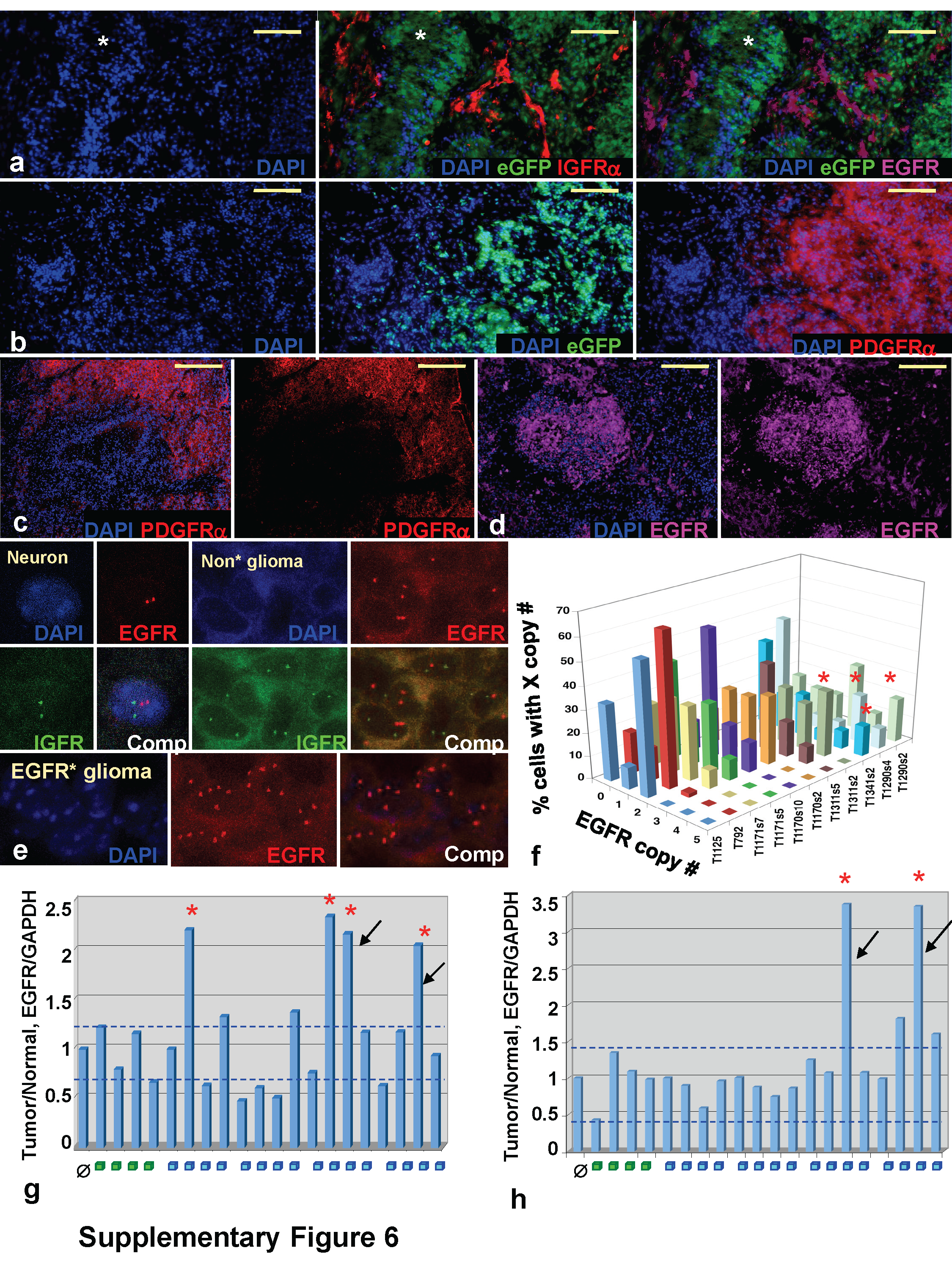

Supplement: Figure S6 — hPDGFb-induced Ntv-a gliomas show regional expression of growth factor receptors important in human glioma biology, expression of which in recruited cells may be associated with low level amplifications in some cases. (a–d) Ntv-a gliomas induced in Ntv-a Ink4a/Arf+/-Pten+/fl mice injected with RCAS-PSG and RCAS-Cre stained with IGFR (red), EGFR (pink) (a,d) or PDGFRα (red) (b,c). Glioma regions predominantly derived from progeny cells (a,b) express PDGFRα; expression of EGFR and IGFR is limited to perivascular areas. (c,d) Adjacent sections stained with PDGFRα and EGFR (red) show regional expression of growth factor receptors; eGFP omitted for easier view. (e) FISH for IGFR (green) and EGFR (red) on Ntv-a GBMs with large areas of recruitment and high EGFR expression. Panels show a neuron, EGFR-expressing non-amplified GBM with extensive recruitment, and EGFR-expressing GBM with EGFR amplification in the recruited cells. (f) Summary of mEGFR FISH performed on EGFR-expressing GBMs with large areas of recruitment. Gliomas containing more than 15% of cells with ≥3 mEGFR signals are marked with an asterisk (amplified). (g,h) real-time qPCR on DNA extracted from sections of mouse Ntv-a GBMs containing large areas of recruitment (blue squares) highly expressing EGFR. Primers for mEGFR kinase domain (g) or intron region of mEGFR (h) were designed by Roche; graphs show fold increase over normal EGFR copy number (∅); gliomas with large areas composed of progeny cells (green squares) were used to establish range of variability of primer noise. Gliomas marked with asterisks were considered amplified, and gliomas marked with arrows appeared amplified by both primer pairs, potentially representing tumors with larger chromosomal amplifications. (TIF) [file pone.0020605.s006.tif]

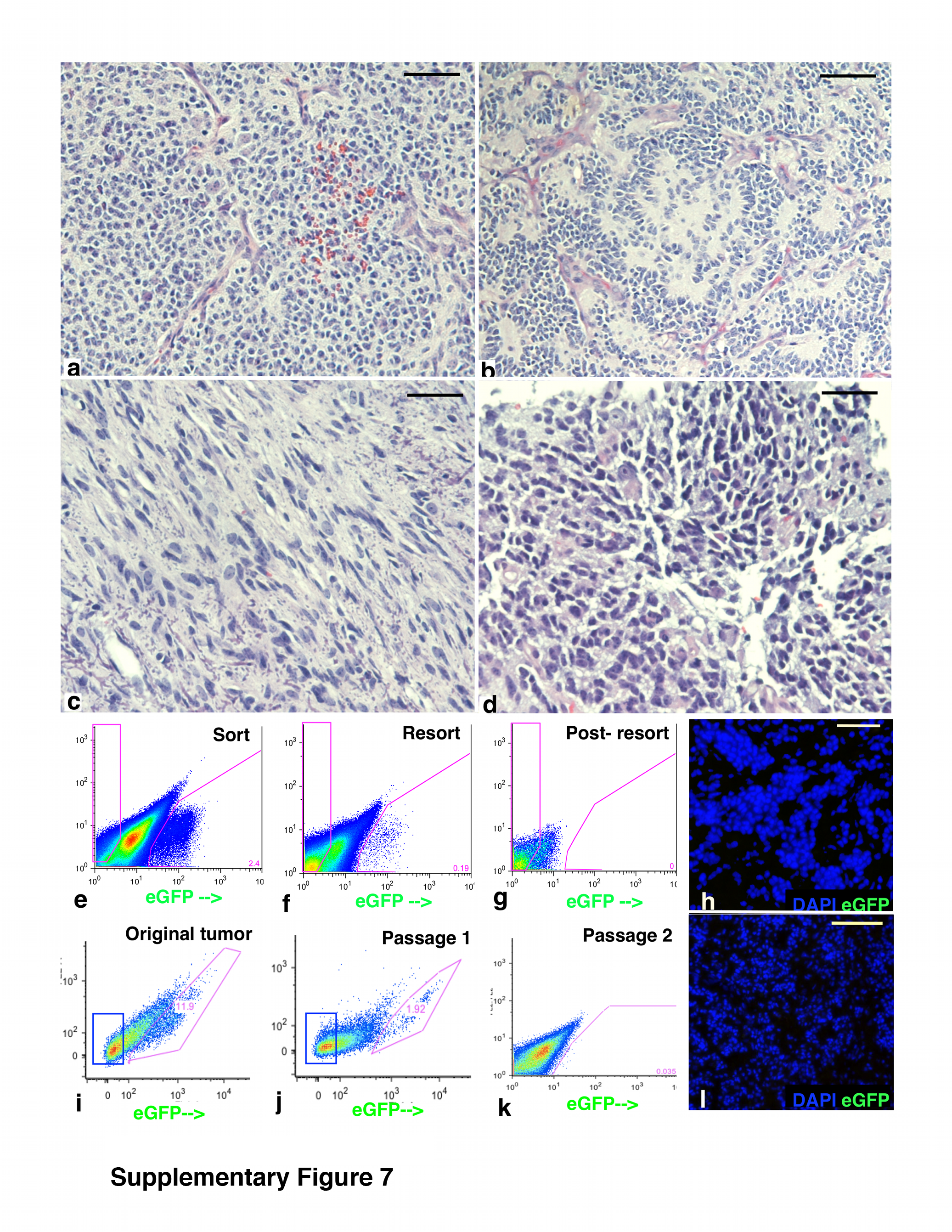

Supplement: Figure S7 — Recruited cells can initiate gliomas upon transplantation and can be serially passaged in mouse hosts. (a–d) H&E-stained sections depicting histology of mouse gliomas induced by transplanting recruited cells. (e–g) Purification of recruited cells from Ntv-a Ink4a/Arf+/-Pten+/fl mice injected with RCAS-PSG and RCAS-Cre; (h) DAPI-stained frozen section of a glioma induced by recruited cells; note absence of cells with virally encoded eGFP. (i–l) Gliomas induced by the recruited cells could be serially passaged in mice. (i–k), FACS plots of gliomas induced by serial transplantation of recruited cells into mouse hosts at passaging; recruited glioma cells were single purified between passages. (l) DAPI-stained section of a transplanted glioma induced by recruited cells, showing absence of progeny cells with virally encoded eGFP. (TIF) [file pone.0020605.s007.tif]

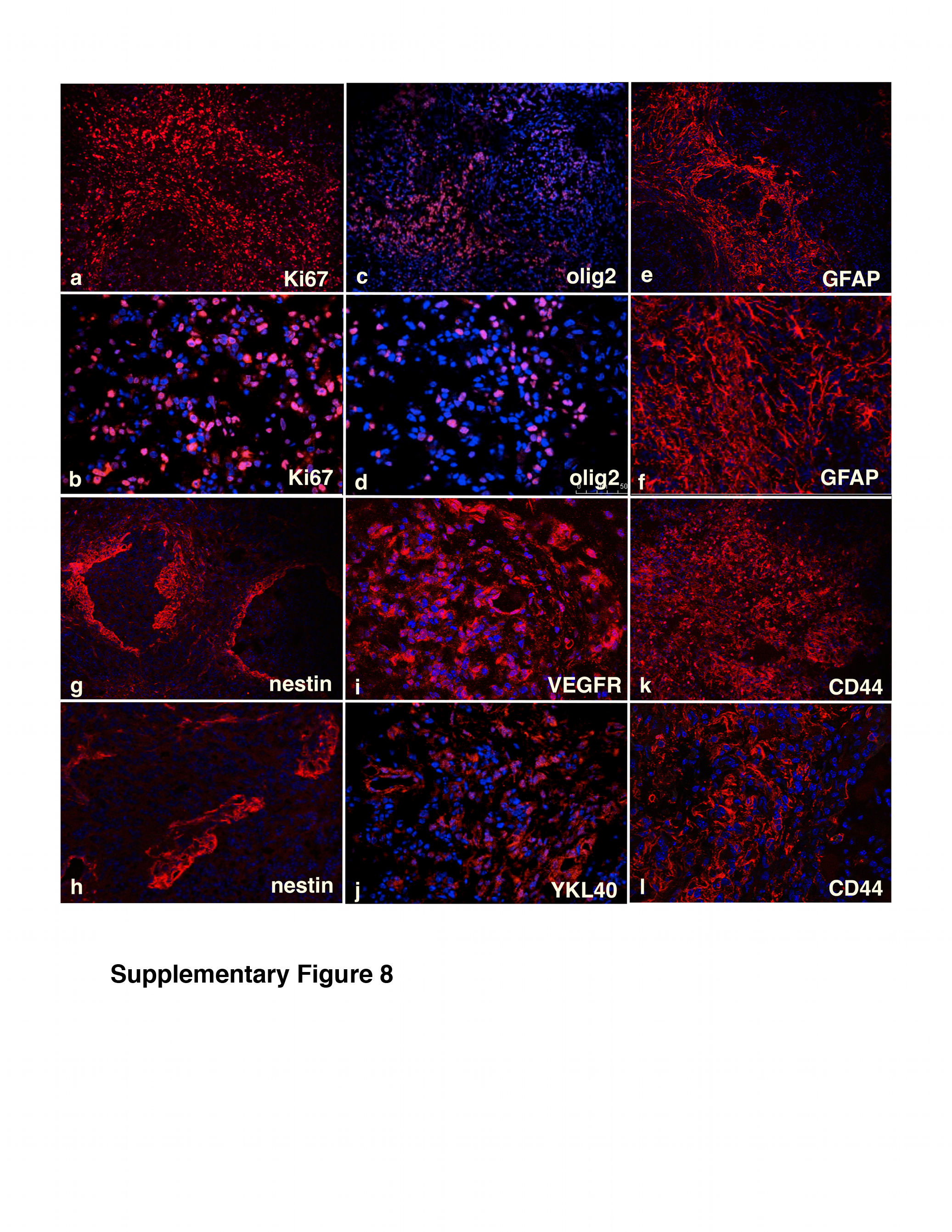

Supplement: Figure S8 — Immunohistochemical analysis of murine gliomas induced by transplanting recruited cells. Images (a–l) show native eGFP and immunostaining for Ki-67 (a,b), olig2 (c,d), GFAP (e,f), nestin (g,h), VEGFR (i), YKL40 (j) and CD44 (k,l). (TIF) [file pone.0020605.s008.tif]

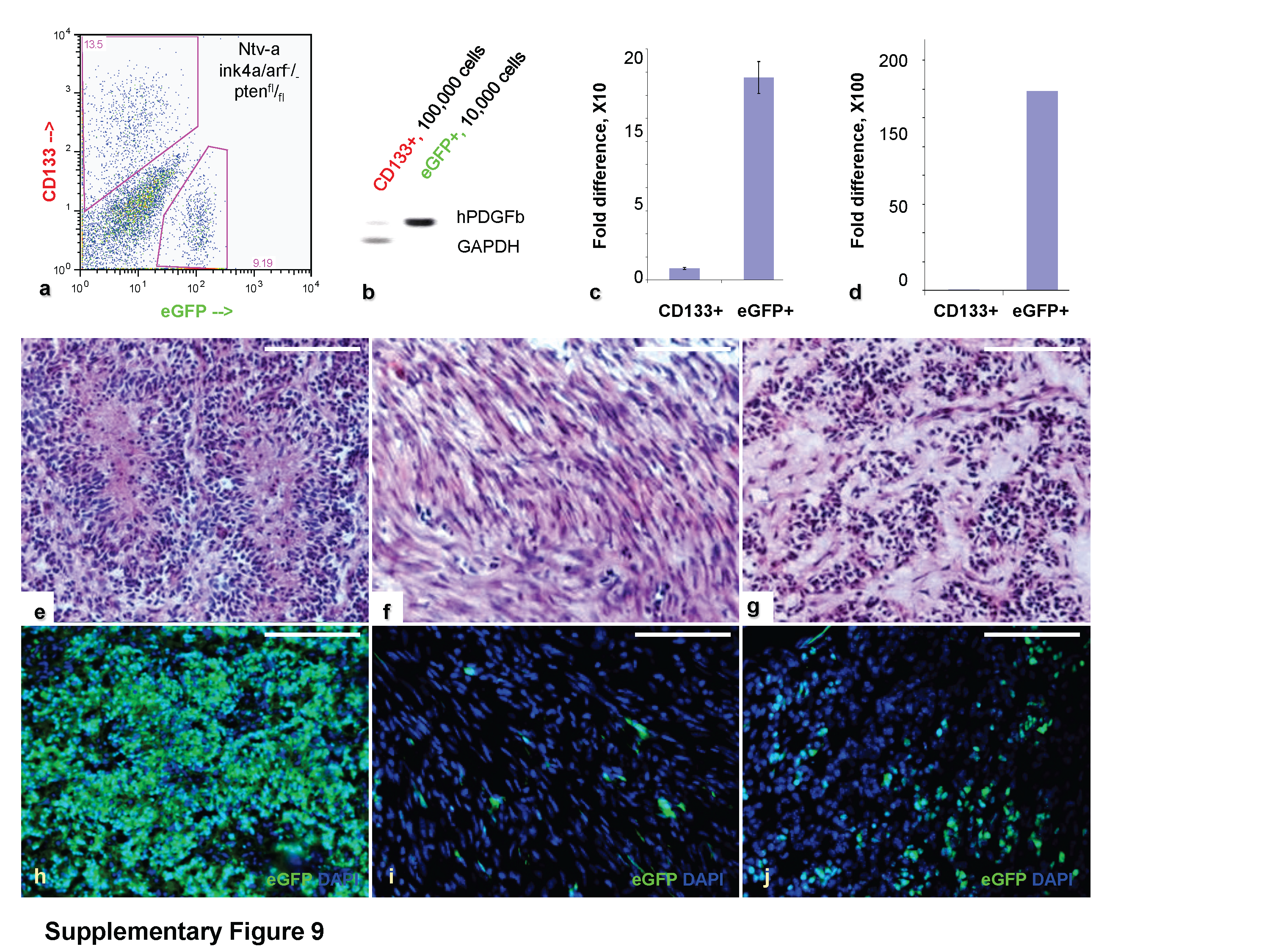

Supplement: Figure S9 — Single-sorted CD133-positive cells are contaminated by low numbers of progeny cells and give rise to gliomas containing small numbers of progeny. FACS plot of an Ntv-a GBM induced by RCAS-PSG and RCAS-Cre, stained with CD133. Single-purified (b,c) CD133-positive cells are recruited, but sorted fractions contain low numbers of progeny cells. Double-purified CD133 cells are recruited (d). (b) PCR and qPCR (c,d) analysis of sorted CD133-positive recruited cells and eGFP-positive progeny, showing absence of viral integration. (e–j) H&E and DAPI-stained sections of transplanted gliomas induced by single-sorted eGFP-positive (e,h) or CD133-positive (f,g,i,j) cells. Note the presence of contaminating progeny in transplanted gliomas induced by single-sorted CD133-positive cell fractions. (TIF) [file pone.0020605.s009.tif]

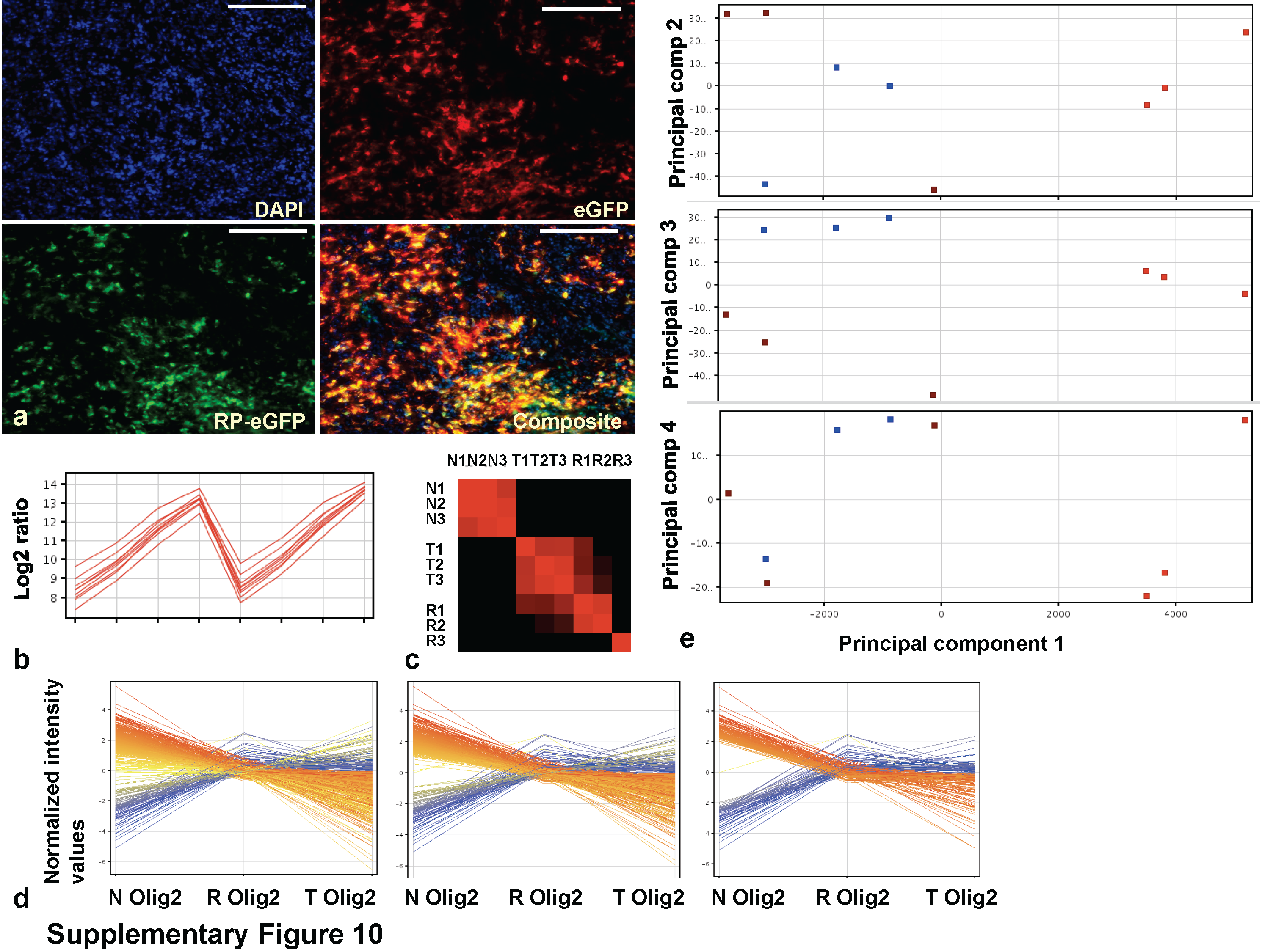

Supplement: Figure S10 — Microarray analysis of normal olig2, recruited olig2 and glioma olig2 cells indicates that recruited cells are similar to glioma cells and differ from normal progenitors. (a) Glioma induced by transplantation of non-fluorescent hPDGFb-driven mouse glioma cells into wild-type olig2 RP-eGFP bacTRAP reporters, showing transgenic RP-eGFP expression and anti-eGFP stain (red). Note large numbers of RP-eGFP-positive recruited cells. (b,c) Quality Controls Metrics plot and Pearson's correlation plot for Affymetrix arrays for normal (N), tumor (T) or recruited (R) olig2 cells. (d) Genespring GX10-generated profile plots showing averaged normalized intensity values for 500 mRNAs most different between glioma and normal olig2 cells (first plot), changing at least two-fold (second plot) or at least five-fold (third plot) between recruited olig2 cells and normal olig2 progenitor cells. (e) Principal component analysis on mRNAs with ANOVA-tested mRNA set removed. Note that all samples do not cluster well when the ANOVA-tested set of mRNAs specifically changed in tumor olig2 cells versus normal olig2 cells is removed. (TIF) [file pone.0020605.s010.tif]

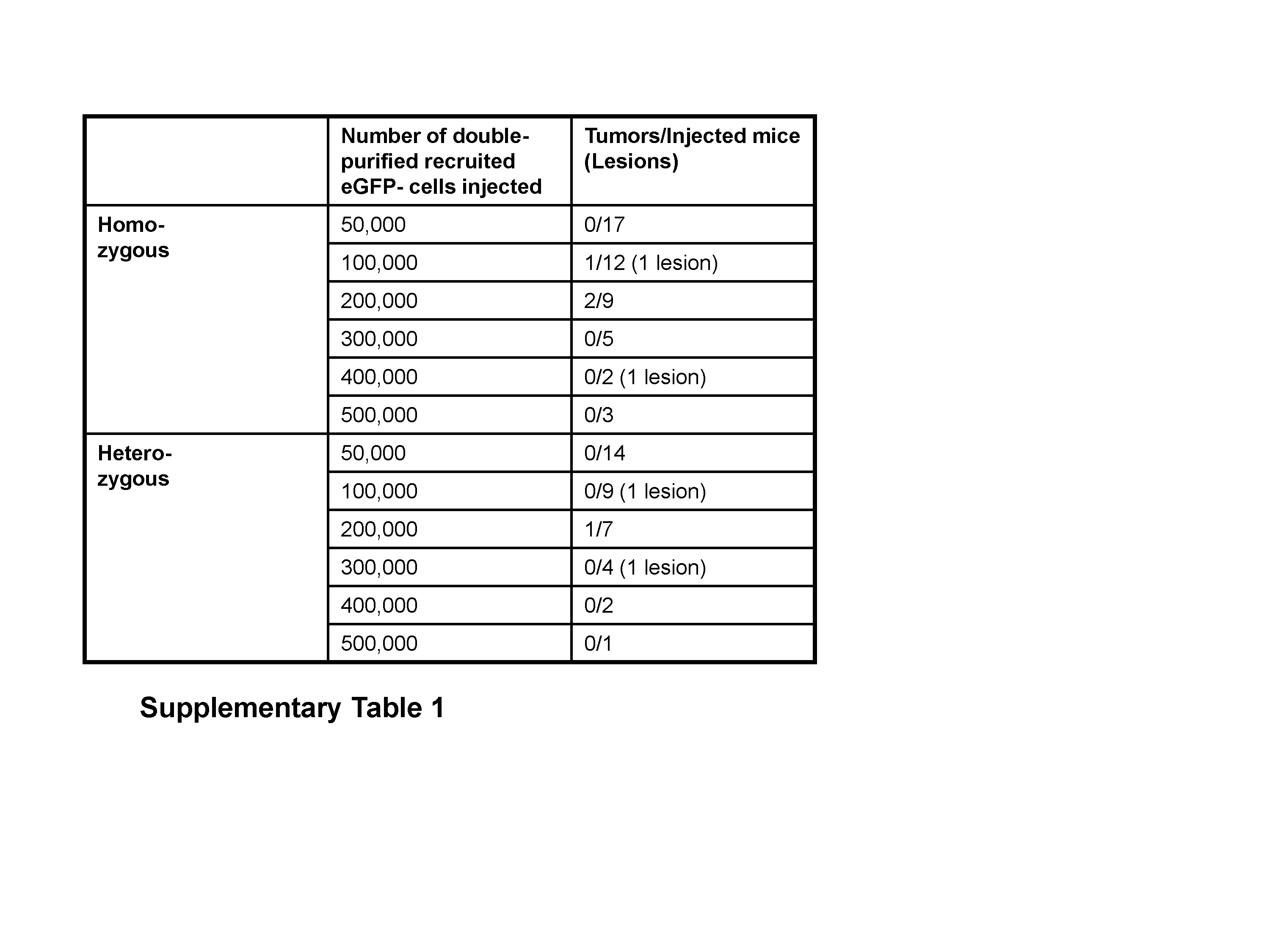

Supplement: Table S1 — The dilution table for transplanted double-sorted recruited eGFP-negative cells. Incidence of gliomas induced by transplanting double-purified eGFP-negative recruited cell fractions derived from Ntv-a RCAS-PSG-induced mouse gliomas homozygous or heterozygous deleted for Ink4a, Arf and Pten at tumor initiation. Shown in the Table are numbers of large tumors and smaller proliferative lesions arising in recipient mice. (TIF) [file pone.0020605.s011.tif]
